# Nanoscale chemical imaging of pseudocapacitive charge storage in MXenes

**DOI:** 10.1039/d5ee05809k

**Published:** 2025-12-16

**Authors:** Namrata Sharma, Louis Godeffroy, Peer Bärmann, Faidra Amargianou, Andreas Weisser, Zoé Dessoliers, Mailis Lounasvuori, Markus Weigand, Tristan Petit

**Affiliations:** a Helmholtz-Zentrum Berlin für Materialien und Energie GmbH, Albert-Einstein-Straße 15 12489 Berlin Germany Tristan.Petit@helmholtz-belin.de; b Faculty of Mathematics and Natural Sciences, TU-Berlin, Hardenbergstraße 36 10623 Berlin Germany; c Faculty III – Process Sciences, TU-Berlin, Straße des 17. Juni 135 10623 Berlin Germany

## Abstract

Pseudocapacitive materials store electrochemical energy through fast and reversible surface charge transfer reactions. Titanium carbide MXenes are two-dimensional materials which have shown redox or intercalation pseudocapacitive properties depending on the electrolyte. Nevertheless, the intrinsic pseudocapacitive charging mechanism in individual MXene flakes remains unresolved. Here, we employ *in situ* scanning transmission X-ray microscopy (STXM) to map the local chemical changes in individual Ti_3_C_2_T_x_ MXene flakes during spontaneous and electrochemical intercalation of protons and lithium ions in aqueous electrolytes. Our investigations reveal that proton and lithium-ion intercalation induces a reduction and an oxidation, respectively, of the titanium atoms in the MXene. This difference reveals a profoundly different chemical origin between redox and intercalation pseudocapacitive processes. By elucidating the interplay between ion hydration, MXene surface chemistry and flake morphology, our study highlights the relevance of chemical imaging in single entities for the fundamental understanding of electrochemical charge storage mechanisms.

## Abstract 2

The intercalation of ions in individual Ti_3_C_2_T_x_ MXene flakes is investigated by scanning transmission X-ray microscopy. We reveal an opposite change in the oxidation state of titanium atoms upon proton and solvated lithium ion intercalation.

## Graphical abstract

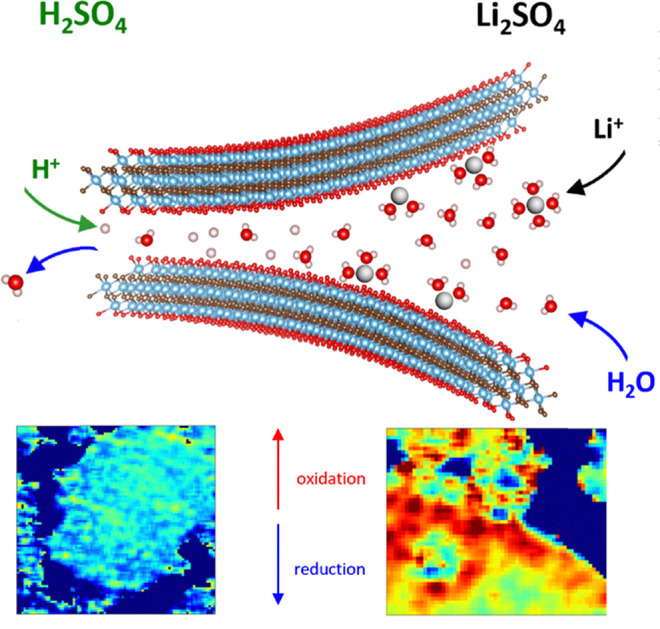

**Broader context**

Pseudocapacitive materials are attracting growing interest because of their ability to reach high energy density while offering faster charging rates and longer stability compared to classical batteries. Two main pseudocapacitive charge storage mechanisms, mainly based on redox reactions or intercalation processes, have been proposed, depending on the pseudocapacitive materials. Two-dimensional transition metal carbides and nitrides, named MXenes, have emerged as an ideal model system to investigate fundamental pseudocapacitive processes under nanoconfinement. In this work, we used synchrotron-based chemical imaging to monitor the local chemical changes in individual titanium carbide MXene flakes during electrochemical cycling in acidic and lithium ion-containing neutral aqueous electrolytes. Our findings show fundamental differences between both electrolytes, with an opposite charge transfer to the titanium atoms upon proton or lithium-ion intercalation. The difference is interpreted in terms of co-intercalation of water molecules with lithium ions, which is not observed in an acidic environment. This result highlights the fundamental role of co-intercalation in a confined environment in pseudocapacitive charge storage.

## Introduction

The increasing demand for high performance energy storage solutions necessitates the development of efficient and fast charging electrochemical systems. Electric double layer capacitors (EDLCs) are attractive because of their ability to charge within seconds and provide nearly unlimited cycle life.^1^ However, their charge storage mechanism relies on non-specific ion adsorption through electrostatic attraction, involving no charge transfer, resulting in a low energy density. In contrast, conventional batteries involving faradaic reactions provide much higher energy density but are hindered by limited power density. The advent of new technologies such as the Internet of Things and wearable electronics calls for a new generation of energy storage devices that can bridge the gap between the fast-charging capability of capacitors and the high energy density of batteries.^2–4^ Pseudocapacitors exhibit these intermediate properties: they store energy through fast surface redox reactions or reversible specific adsorption of ions which do not involve phase transformation of the electrode material.^5^ While pseudocapacitive materials offer a pathway to higher energy densities, the transition from EDLCs to pseudocapacitors still remains under discussion.^6^

Recently, 2D transition metal carbides and nitrides, so-called MXenes, have emerged as a promising model system for understanding pseudocapacitive charge storage mechanisms.^7^ MXenes exhibit a unique combination of a layered structure, surface functionality, nanosized interlayer spacing, and a conductive core that can be finely tuned.^8,9^ Their electrochemical charge storage mechanism varies depending on the electrolyte, involving electrostatic interactions, faradaic reactions, or a combination of both.^10–12^ The current consensus on Ti_3_C_2_T_x_ (where T_x_ represents the surface terminations) in aqueous neutral electrolytes suggests competing contributions from electrical double-layer (EDL) and pseudocapacitive charge storage, driven by the electrostatic interaction between inserted solvated cations and the MXene surface within the confined interlayer spaces.^13^ In contrast, in acidic electrolytes, charge storage is primarily attributed to redox pseudocapacitance *via* fast electrochemical (de)protonation of the oxygenated surface terminations following the intercalation of protons.^14^ This mechanism has been supported by X-ray absorption spectroscopy (XAS) and Raman spectroscopy.^15,16^ However, most studies have focused on macroscale electrodes so far, where the intrinsic ion–host interaction mechanism within individual MXene flakes remains inaccessible due to the non-uniform stacking of MXene sheets. Local inhomogeneity of the ion intercalation in individual MXene flakes remains unexplored experimentally.

A technique with high spatial resolution and high chemical sensitivity allowing chemical mapping at the sub-flake level is required to address this challenge. Soft X-ray microscopy techniques, including X-ray photoemission electron microscopy (X-PEEM)^17,18^ and scanning transmission X-ray microscopy (STXM),^19^ were recently applied to monitor the surface chemistry of single MXene flakes. The bulk sensitivity of STXM is particularly suited to monitor the MXene surface chemistry within the interlayer spacing. We have recently applied *in situ* STXM to MXenes in an aqueous electrolyte, showing that the MXene surface chemistry is strongly affected by intercalating species.^20^*In situ* electrochemical STXM has also been proven to be an ideal technique for tracking ion intercalation in battery materials.^21^ Enabling XAS with a sub-50 nm spatial resolution, STXM, for instance, allowed mapping of the local phase-transformation mechanism of Li_*x*_FePO_4_ during electrochemical (de)intercalation in solid^22^ and liquid electrolytes.^23^

Here, we employ *in situ* electrochemical STXM to investigate the local chemical changes within individual Ti_3_C_2_T_x_ MXene flakes upon spontaneous and electrochemical (de)intercalation of H^+^ and Li^+^ cations in aqueous electrolytes. The high chemical sensitivity at the Ti L-edge and the spatial resolution down to 50 nm in a liquid environment enable the mapping of the titanium oxidation state over individual flakes of thicknesses varying between 2 and 35 MXene layers (Supplementary Discussion 1). Our findings reveal that Ti atoms in Ti_3_C_2_T_x_ MXenes undergo reduction upon H^+^ intercalation in 0.1 M H_2_SO_4_, but oxidation upon Li^+^ intercalation in 0.1 M Li_2_SO_4_, challenging the current molecular picture of pseudocapacitive charging in Ti_3_C_2_T_x_ MXenes in aqueous electrolytes. Furthermore, we explore the influence of the MXene flake thickness and local inhomogeneities in the surface chemistry at the sub-flake level on intercalation processes. This study provides novel insights into the fundamental mechanisms underlying pseudocapacitive charging in Ti_3_C_2_T_x_ MXene flakes at the nanometre scale, under both acidic and neutral conditions.

## Results and discussion

**Fig. 1:**
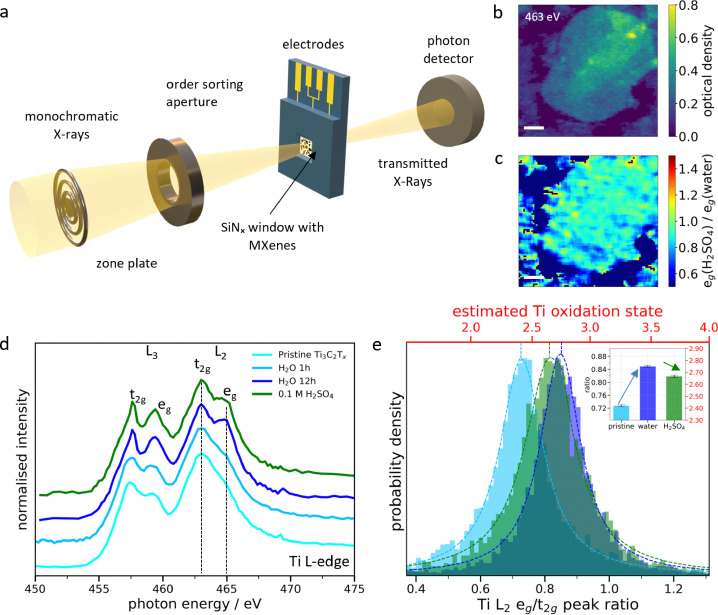
**Spontaneous H^+^ intercalation in individual Ti**_**3**_ **C**_**2**_ **T**_**x**_ **MXene flakes**. (a) Schematic of the *in situ* STXM setup. (b) STXM image of a few-layered Ti_3_C_2_T_x_ MXene flakes acquired at the Ti L-edge (463 eV) in water. (c) Heat map of the intensity ratio of the Ti L_2_ e_g_ peak in 0.1 M H_2_SO_4_ to that in water (e_g_(H_2_SO_4_)/e_g_(H_2_O)) show localised chemical changes in the Ti oxidation state upon electrolyte exchange. A ratio below (above) 1 indicates a relative reduction (oxidation) of the probed Ti atoms (Fig. S9). (d) Average XAS spectra of all MXene flakes in (a) in their pristine state (light blue), after 1 h of exposure to water (blue), after 12 h of exposure to water (dark blue), and subsequent exposure to 0.1 M H_2_SO_4_ (green). (e) Pixel intensity histograms of the Ti L_2_ e_g_/t_2g_ peak ratio heat maps (Fig. S8) with the estimated Ti oxidation state on the top axis (Supplementary Discussion 2 and Fig. S6). Scale bars in (b) and (c) are 1 µm.

Ti_3_C_2_T_x_ MXenes were synthesized by wet chemical etching of the Ti_3_AlC_2_ MAX phase using a mixture of HCl and HF and delaminated as previously reported (see Methods),^24^ leading to micrometer-sized few-layered Ti_3_C_2_T_x_ MXene flakes (Fig. S1–S3). *In situ* STXM was performed using an ultra-corrosion resistant transmission flow cell holder in which MXene flakes and a thin electrolyte layer are sandwiched between two X-ray transparent windows (Fig. 1a and Fig. S4). For electrochemical measurements, carbon, Au or Pt microelectrodes were deposited on one of the windows. The flow cell configuration allows exchanging the electrolyte and monitoring the associated changes in the electronic structure of the Ti atoms *via* the change of the absorption at the Ti L-edge. The local changes in MXene surface chemistry upon exposure to water, 0.1 M H_2_SO_4_ and 0.1 M Li_2_SO_4_ are described as follows.

### Spontaneous H^+^ intercalation in individual Ti_3_C_2_T_x_ MXene flakes

MXene flakes with thicknesses ranging from 2 to 14 layers (as estimated from their optical density, see Fig. 1b and Fig. S7) were first exposed to water, followed by 0.1 M H_2_SO_4_. XAS at the Ti L-edge (Fig. 1d) probes the electronic transitions from the Ti 2p_3/2_ and 2p_1/2_ core levels to the 3d orbitals, giving rise to the L_3_ and L_2_ edges.^25,26^ Crystal field splitting of the 3d orbitals induces a second splitting between the t_2g_ and e_g_ orbitals for both L_3_ and L_2_ edges, whose relative intensity is related to the density of unoccupied d-states of the Ti atoms and therefore to their oxidation state. The mean Ti L_2_ e_g_/t_2g_ peak intensity ratio is used to estimate the average oxidation state of the Ti atoms in the MXenes (see Supplementary Discussion 2 and Fig. S6).^18,19^

Water exposure for 1 hour did not show any changes in the spectral features, as shown in Fig. 1d. However, 12 hours of exposure results in oxidation of the Ti atoms, with a change in the estimated average Ti oxidation state from 2.40 to 2.78 (Fig. S6) evidenced by the evolution of the Ti L_2_ e_g_-edge (Fig. 1d and e). The water-induced oxidation observed in individual Ti_3_C_2_T_x_ MXenes may be related to the first steps of MXene hydrolysis.^20,27^

Subsequent exposure of the MXene flakes to 0.1 M H_2_SO_4_ for one hour shows that the water-induced Ti oxidation is partially reversible. The evolution of the Ti L-edge XAS spectra (Fig. 1d) indeed highlights a decrease of the e_g_ peak. The thickness dependent Ti oxidation state changes are presented in Fig. S7 which shows a fall in the Ti-L_2_ e_g_/t_2g_ around 0.05 for all flake thicknesses. This suggests that the broad distribution of the Ti-L_2_ e_g_/t_2g_ peak ratio (Fig. 1e and Fig. S8) is related to local variations of the Ti-chemical bonding over the flake rather than the thickness. The L_2_ e_g_ ratio heat map between H_2_SO_4_ and water (Fig. 1c and Fig. S9) enables a direct chemical imaging of the regions with reduced Ti atoms at the flake level. This chemical reduction is non-homogeneous over the flake. This may be related to a non-uniform surface chemistry composed of a mixture of O, OH and F-terminations or defects.^28,29^ Since H^+^ ions are most likely to protonate O-terminated sites, the latter may be located in regions where the e_g_ ratio is <1, while regions where the e_g_ ratio ≈ 1 might be related to locally higher inactive F terminations. To test this hypothesis, we have estimated the percentage of active pixels (e_g_ ratio is <1) for both thin (OD < 0.35, ∼9 layers and below) and thick flakes (OD > 0.35, above ∼9 layers) using the e_g_ ratio heatmaps (Supplementary Discussion 3 and Table S2) and compared them to XPS measurements. We found approximately 73% active pixels on thin flakes and 93% on thick ones, which corresponds well to the amount of F terminations quantified by XPS (Fig. S3).

In our study, we believe that the protonation of active oxygen terminations displaces adsorbed water molecules – as protonation is energetically more favorable – reducing the electron density around the oxygen atoms and hence decreasing the Ti oxidation state.^30^ Notably, although the water-induced oxidation is not fully reversible, no alteration of the flake structure was observed, such as TiO_2_ particle formation which is commonly reported.^31^

### Electrochemical H^+^ intercalation in individual Ti_3_C_2_T_x_ MXene flakes

**Fig. 2:**
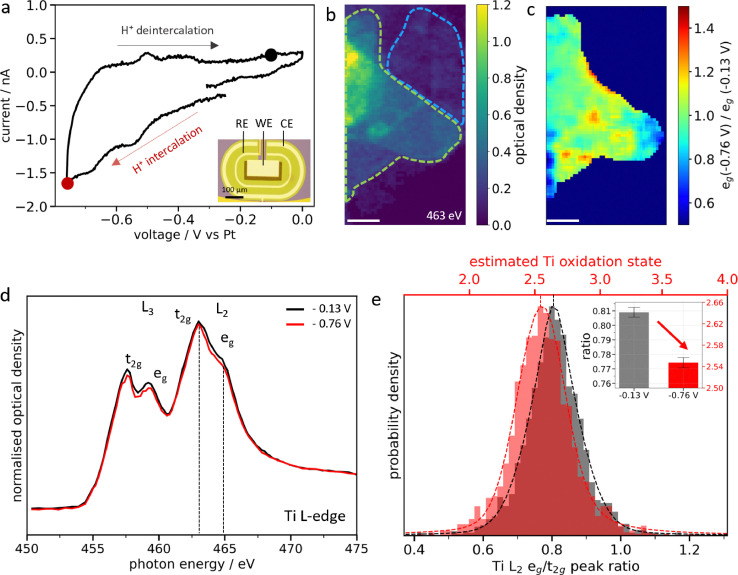
**Electrochemical H** ^**+**^ **intercalation in individual Ti**_**3**_ **C**_**2**_ **T_x_ MXene flakes**. (a) Cyclic voltammogram of Ti_3_C_2_T_x_ MXenes in 0.1 M H_2_SO_4_ measured using a 3-electrode electrochemical cell with a carbon working electrode and Pt counter and reference electrodes. Inset: optical image of an electrochemical chip showing the electrode geometry. (b) STXM image of Ti_3_C_2_T_x_ flakes measured at the Ti L-edge (463 eV) in 0.1 M H_2_SO_4_ on the chip mentioned in (a). The region of interest consists of thick overlapping few-layered MXene flakes (green) surrounded by bi-layered MXene flakes (blue). (c) Heat map of the intensity ratio of the L_2_ e_g_ peak at −0.76 V and −0.13 V *vs*. Pt (e_g_(−0.76 V)/e_g_(−0.13 V)). (d) XAS spectra averaged over all flakes in (b), at −0.13 V and −0.76 V *vs*. Pt, respectively. (e) Pixel intensity histograms of the L_2_ e_g_/t_2g_ peak ratio heat maps (Fig. S12) at these two potentials over all flakes shown in (c). Inset: estimated average Ti oxidation state. Scale bars in (b) and (c) are 1 µm.

Following spontaneous intercalation in 0.1 M H_2_SO_4_ electrolyte, the electrochemical H^+^ (de)intercalation was monitored by applying an electrical potential in a 3-electrode setup (Fig. 2a). Cyclic voltammetry shows the characteristic pseudocapacitive response of Ti_3_C_2_T_x_ MXenes, with large reversible redox peaks centered around −0.5 V *vs*. Pt (Fig. 2a).^8^ The chemical imaging of the potential-induced change in MXene chemistry was performed on few-layered overlapping flakes with thicknesses ranging from 2 to 23 layers at OCV (−0.13) and applied −0.76 V (Fig. 2b and Fig. S10). The average oxidation state of the Ti atoms is found to depend on the thickness of the flake (Fig. S10 and S11). For bi-layered flakes (blue dashed region in Fig. 2b), no significant change is observed under applied potential. However, for thick overlapping flakes (green dashed region in Fig. 2b), a subtle decrease in the L_2_ e_g_ peak intensity at −0.76 V *vs*. Pt is observed compared to that at −0.13 V (Fig. 2d and Fig. S11), leading to an average L_2_ e_g_/t_2g_ peak ratio decrease from 0.81 to 0.77 at −0.76 V (Fig. 2e and Fig. S12). This corresponds to a reduction in the Ti oxidation state of ∼0.1 e^−^ per atom and confirms the redox pseudocapacitance charging mechanism. The specific capacitance in 0.1 M H_2_SO_4_ is estimated to be 370 F g^−1^ (see Supplementary Discussion 4), which is close to the state-of-the-art reported for microporous templated electrodes (380 F g^−1^) due to the high accessibility of redox sites with single MXene flake.^32^ Furthermore, the e_g_ peak ratio heat map calculated between −0.76 V and −0.13 V for the thick flakes (Fig. 2c and Fig. S13) shows uneven distribution of the Ti oxidation state over the MXene flake. This corresponds to 64% of the active area in thin flakes and 88% in thick flakes (Supplementary Discussion 3 and Table S2), and this reduction in the active area for thin flakes could be due to water-induced oxidation. Local hotspots of higher Ti oxidation (inactive areas) are identified over the flake, which are more intense than the ones observed for spontaneous intercalation (Fig. 1).

### Spontaneous Li^+^ intercalation in individual Ti_3_C_2_T_x_ MXene flakes

**Fig. 3:**
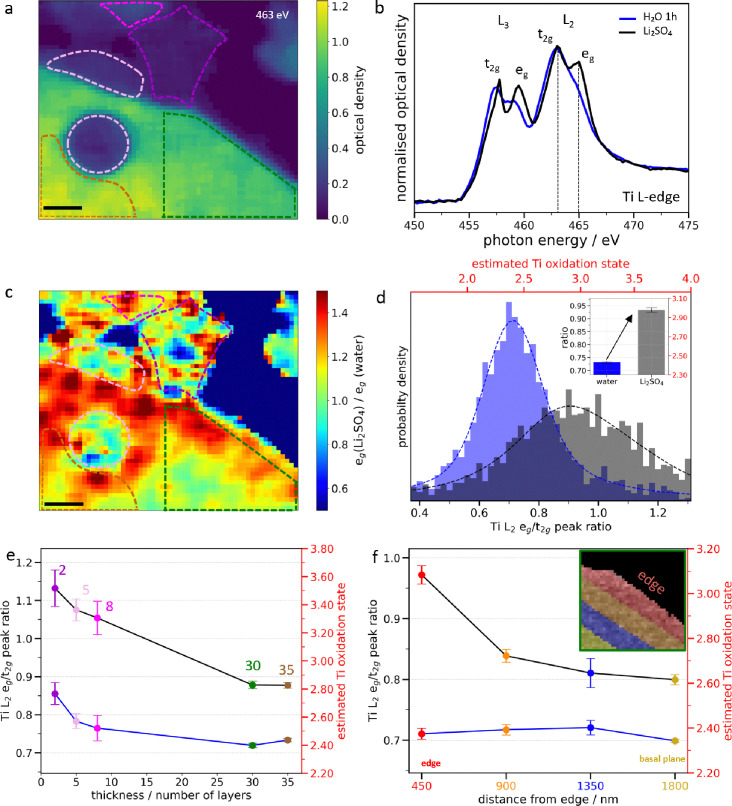
**Spontaneous Li** ^**+**^ **intercalation in individual Ti**_**3**_ **C**_**2**_ **T_x_ MXene flakes**. (a) STXM image at the Ti L-edge (463 eV) in water. Highlighted regions correspond to thicknesses of 2 (purple dashed), 5 (pink dashed), 8 (light purple dashed), 30 (green dashed) and 35 layers (yellow dashed), respectively. (b) XAS spectra averaged over all flakes in water (blue) and 0.1 M Li_2_SO_4_ (black), respectively. (c) Heat map of the intensity ratio of the L_2_ e_g_ peak in 0.1 M Li_2_SO_4_ and water (e_g_(Li_2_SO_4_)/e_g_(H_2_O)) (Fig. S18). (d) Pixel intensity histograms of the L_2_ e_g_/t_2g_ peak ratio heat maps (Fig. S15) in water (blue) and 0.1 M Li_2_SO_4_ (black), respectively. (e) Thickness-dependent evolution of the estimated Ti oxidation state, and (f) edge-to-basal plane variation of the estimated Ti oxidation state in water (blue line) and 0.1 M Li_2_SO_4_ (black line), respectively. Inset: Regions divided from the edge to the basal plane in the 30-layered flake. Scale bars in (a) and (c) are 1 µm.

A similar methodology was applied to monitor Li^+^ ion intercalation in few-layered Ti_3_C_2_T_x_ MXene flakes with thicknesses ranging from 2 to 35 layers in an aqueous neutral 0.1 M Li_2_SO_4_ electrolyte (Fig. 3a and Fig. S14).^19^ The XAS spectra at the Ti L-edge averaged over the flakes in Fig. 3a exhibit a prominent splitting of the Ti L_2_-edge (Fig. 3b) corresponding to an increase of the estimated Ti oxidation state of +0.55 after electrolyte exchange (Fig. 3d and Fig. S15). A similar change of the XAS spectra was previously reported under spontaneous intercalation in macroscopic MXene films.^33^ Similar results were also obtained in 0.1 M LiCl (Fig. S16), suggesting that the anions probably do not intercalate and hence contribute to the Ti oxidation, as suggested by previous gravimetric studies.^34^ Furthermore, the broad distribution of the L_2_ e_g_/t_2g_ peak ratio values suggests a non-uniform oxidation of the MXenes in the Li^+^ environment. Indeed, we see that the Ti oxidation state scales with the flake thickness, with the thinnest flakes being more oxidized (Fig. 3e and Fig. S17). Furthermore, the e_g_ peak ratio heat map (Fig. 3c) highlights regions of localized oxidation, showing approximately 93% active sites on thick flakes (>9 layers) compared to 73% on thin flakes (≤9 layers) (Supplementary Discussion 3 and Table S2). The lower fraction of active sites in thin flakes further indicates that these flakes are more prone to water induced oxidation.

Additionally, for thick MXene flakes, a gradient of the Ti oxidation state from the edge to the basal-plane is clearly visible (Fig. 3c, f and Fig. S17, S18), as quantitively monitored with the L_2_ e_g_/t_2g_ peak ratio (Fig. 3f). Flake edges may be more reactive due to a higher defect density and more extensive exposure to the electrolyte, which together results in stronger oxidation of the flake edges than the basal plane upon cation intercalation. In addition, the MXene flakes might also become stiffer with thickness.^35^ As the thickness of the flake increases, the solvated ions and water molecules might become less able to intercalate deeply within the basal plane. In contrast, interaction with the surface of the MXene flake is not limited by ion diffusion within the interlayer and hence the homogeneous Ti oxidation observed on thin flakes. As the number of layers increases, the contribution of the top layer also decreases and thus a lower increase in the Ti oxidation state is witnessed. Although we cannot distinguish this oxidation process from that induced by prolonged exposure to water, it occurs much faster (tens of minutes vs several hours), so free water molecules most likely play a minor role in this case. Overall, these findings suggest that Li^+^ ion intercalation is thickness-dependent and leads to an oxidation of Ti atoms, in contrast to proton intercalation where a reduction is observed.

### Electrochemical Li^+^ intercalation in individual Ti_3_C_2_T_x_ MXene flakes

**Fig. 4:**
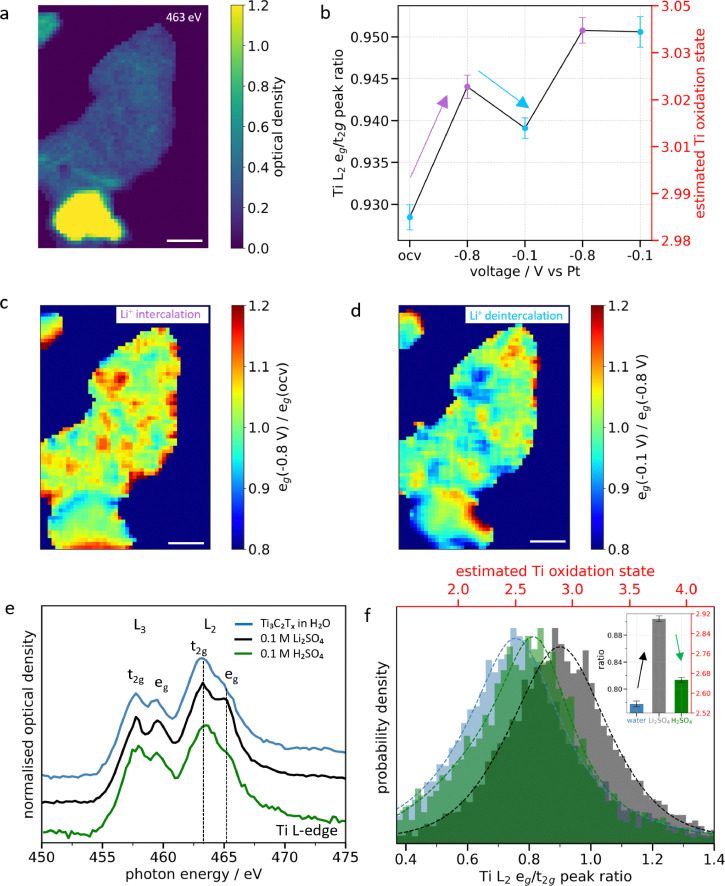
**Electrochemical intercalation of Li** ^**+**^ **in individual Ti**_**3**_ **C**_**2**_ **T**_**x**_ **MXene flakes**. (a) STXM image at the Ti L-edge (463 eV) in 0.1 M Li_2_SO_4_ with ∼6- and 15-layer flakes. (b) Comparative Ti L_2_ e_g_/t_2g_ peak intensity ratios in the charged and discharged states over two consecutive cycles. (c) and (d) Heat map of the intensity ratio of the L_2_ e_g_ peak between −0.8 V *vs*. Pt and the OCV (e_g_(−0.8 V)/e_g_(OCV)) for intercalation and between −0.1 V and −0.8 V (e_g_(−0.1 V)/e_g_(−0.8 V)) for deintercalation, respectively. (e) XAS spectra averaged over all flakes in water (blue), 0.1 M Li_2_SO_4_ (black) and 0.1 M H_2_SO_4_ (green), respectively. (f) Corresponding pixel intensity histograms of the L_2_ e_g_/t_2g_ heat maps (Fig. S23). Scale bars in (a), (c) and (d) are 1 µm.

The electrochemical Li^+^ ion (de)intercalation was monitored in the 0.1 M Li_2_SO_4_ electrolyte on a few-layered Ti_3_C_2_T_x_ MXene flake (Fig. 4a and Fig. S19). The cyclic voltammetry shows the characteristic capactive response of Ti_3_C_2_T_x_ MXenes with no redox peaks (Fig. S19b). As shown in Fig. 4b, the Ti atoms further oxidize upon electrochemical Li^+^ intercalation corresponding to an increase in the Ti oxidation state of ∼0.04 e^−^ per atom, although an overall reduction of (negative) current is measured (at least during the first cycle, see Fig. S20). The oxidation state change is related to a specific capacitance of 147 F g^−1^ (Supplementary Discussion 4), which is slightly lower than the capacitance reported for bulk electrodes.^36^ In that case, the high accessibility of the redox sites of single MXene flakes may be detrimental compared to stacked films, because the irreversible water-induced oxidation occurring in the first 2–3 cycles may be more. Conversely, the Ti atoms are reduced upon Li^+^ deintercalation while an overall oxidation (positive) current is measured. This counterintuitive behavior, in contrast to the one observed in H_2_SO_4_, points to a more complex redox mechanism which does not involve the Ti atoms directly. Additionally, the effect of the flake thickness on Ti oxidation is analyzed (Fig. S19f). Similar behavior is observed over two cycles, but the extent of change is greater in thin flakes compared to thick ones, consistent with the trend observed during spontaneous intercalation.

The e_g_ ratio heat maps presented in Fig. 4c and d (and Fig. S21) show non-uniform oxidation and reduction across the flakes. Both thin and thick flakes exhibit approximately 70% electrochemically active area during oxidation, which decreases to around 60% during reduction. This decrease in active sites likely results from irreversible oxidation caused by a combination of surface lithiation and water-induced oxidation. Similar to spontaneous intercalation, thinner flakes exhibit localized “hotspots” of activity across the basal plane, likely associated with surface defects or inhomogeneous surface chemistry.^37^ In contrast, thicker flakes exhibit activity primarily along their edges (Fig. S19c and d). As discussed above, the increased stiffness of thicker MXene flakes may confine their activity to the edges, and although similar surface defects are likely still present, their contribution to the overall Ti signal becomes negligible in transmission.

As shown in Fig. 4b, the trend remains consistent across two consecutive cycles, even if some degree of irreversible oxidation is observed. This might be due to incomplete Li^+^ deintercalation as observed by Xie *et al.*^38^ To force ion deintercalation and reduce the MXenes, a higher positive potential of +0.2 V *vs*. Au was applied in a second experiment (Fig. S19f). While this slightly reduced the Ti atoms in the MXenes, it still did not restore their initial oxidation state. When an even higher positive potential of +0.6 V *vs*. Au was applied (Fig. S22), irreversible Ti oxidation occurred, suggesting that the potential required to fully de-intercalate the ions exceeds the oxidation potential of Ti_3_C_2_T_x_ MXenes, making complete electrochemical reversibility challenging in dilute aqueous electrolytes. In contrast, the MXene flakes were observed to recover almost their pristine oxidation state in 0.1 M H_2_SO_4_ (Fig. 4e, f and Fig. S23).

### Discussion

**Fig. 5:**
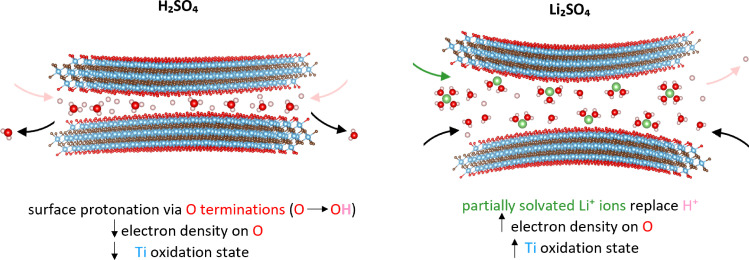
Schematic view of proton and Li^+^ ion intercalation within Ti_3_C_2_T_x_ MXenes in the aqueous electrolyte. In the acidic electrolyte (left), proton intercalation displaces confined water molecules, protonating the MXene surface, which results in a reduced Ti oxidation state. In the neutral electrolyte containing Li^+^ (right), the interaction of partially desolvated Li^+^ ions and water with the MXene surface results in an increased Ti oxidation state.

By revealing different redox processes that occur in Ti_3_C_2_T_x_ MXenes during the intercalation of H^+^ and Li^+^ ions into single few-layered flakes in aqueous electrolytes, *in situ* STXM extends our understanding of pseudocapacitive processes in MXenes as presented schematically in Fig. 5. In an acidic environment, the intercalation of protons results in the reduction of the Ti atoms, likely due to the protonation of O-terminal groups.^15^ Conversely, the average Ti oxidation state of the MXenes increases upon Li^+^ intercalation.

This contrasting behaviour may arise from differences in ion hydration and related chemical interactions between co-intercalated water and the MXene surface. As explained by the Grotthuss mechanism,^39^ H^+^ ions lack a well-defined hydration shell, allowing close interactions with the electron dense –O terminations. Comparatively, Li^+^ ions have a stronger solvation shell, even though they are partially desolvated upon intercalation due to the 2D confinement in the interlayer spacing.^40^ Two chemical interactions may then occur. Firstly, the co-intercalating water molecules might oxidize directly the MXene surface as observed in our prolonged water exposure measurements (Fig. 1d and e). The oxidation induced by prolonged water exposure leads to hydrolysis. Water molecules can interact with the Ti sites in MXene layers, particularly at defect sites or edges, facilitating oxidation. For example, Doo *et al.*^41^ demonstrated that the oxidative degradation of Ti_3_C_2_T_x_ in aqueous dispersions is accelerated at elevated temperatures, with water acting as the medium for oxygen transfer and proton exchange. Similarly, Xia *et al.*^42^ showed *via* scanning transmission electron microscopy studies that oxygen from water molecules preferentially attacks under-coordinated Ti atoms at edges or defects, initiating oxidation even in the absence of additional oxidants.

However, this process is generally slow and largely surface limited.^27,43^ The presence of co-intercalated water within the MXene interlayers, as shown by *in situ* STXM when the flakes are exposed to Li^+^ ions,^20^ can increase the local mobility of reactive species, enhancing oxidation at sites that are otherwise less accessible.^44^ This effect is distinct from simple surface wetting, as co-intercalated water can penetrate deeper into the flake structure, exposing more Ti atoms to hydrolytic attack.^45^

Taken together, these studies support the idea that co-intercalated water contributes directly to Ti oxidation, at a much faster rate than the oxidation caused by prolonged exposure to water in the absence of Li^+^. In our experiments, short-term exposure (1 hour) to pure water shows minimal spectral changes, whereas longer-term water exposure (12 hours) leads to partially irreversible Ti oxidation (Fig. 1d). However, in the case of Li^+^ ion intercalation, oxidation occurs much faster, suggesting a direct chemical interaction between the Li^+^ ions and the MXene surface. In Li_2_SO_4_, hydrophilic Li^+^ ions co-intercalate with water molecules, enabling access to deeper sites and thus increasing the Ti oxidation state even within 1 hour. Similar results were recently reported in chlorine electrolytes with other cations.^20^ This process is more reversible in H_2_SO_4_ (Fig. 4e) compared to water induced oxidation, consistent with the mechanistic understanding that ion-water co-intercalation promotes transient, reversible Ti oxidation.

Interestingly, the Ti oxidation state changes induced by electrochemical Li^+^ intercalation are only observed over 2–3 cycles (Fig. 4b), after which the Ti oxidation state remains constant, but can still be reduced in H_2_SO_4_ afterwards. Most likely, after spontaneous intercalation and few cycles of electrochemical intercalation, the adsorption sites for desolvated Li^+^ ions are saturated and hydrated Li^+^ ions (de)intercalate without involving faradaic charge transfer anymore, transitioning from redox to purely capacitive behaviour as typically reported in the literature.^6^

The role of the surface terminations is crucial in explaining the observed Ti oxidation state changes. While –OH and –F groups tend to stabilize the MXene surface, –O terminations tend to act as highly active redox sites due to their high electron density. A direct correlation between surface terminations and the number of active pixels is therefore expected. The high average active area during spontaneous intercalation (70–90%) can be attributed to the surface termination distribution. XPS analysis indicates that the MXenes contain approximately 30–40% –F terminations (Fig. S3), which are inactive and might account for the observed fraction of the inactive area. After spontaneous intercalation, applying a negative potential in both electrolytes further drives intercalation. Since some sites are already protonated or lithiated due to spontaneous intercalation, the average number of active sites decreases by roughly 10%.

Bo *et al.* showed strong interactions between surface oxygen and intercalated Li^+^ ions using TEM and molecular dynamic simulations.^46^ Additionally, co-intercalating water molecules can hydrate the protons on the –OH terminations, weakening the O–H bond. Partially desolvated Li^+^ ions can then easily replace H^+^ ions on the –O terminations.^47^ As Li^+^ ions occupy these active areas, they replace protons that normally help screen the electron dense –O terminations.^48^ Since Li^+^ has a lower effective charge density due to its solvation shell, replacing H^+^ reduces the charge screening efficiency. Consequently, the electron-withdrawing effect of –O termination on Ti increases, raising the oxidation state of underlying Ti atoms. We believe that the combined effect of both Li^+^ ion and water interaction results in an increased Ti oxidation state. Moreover, applying a cathodic potential of >+0.6 V *vs*. Au irreversibly oxidizes the MXene, highlighting the constraints posed by the narrow electrochemical stability window of MXenes in aqueous electrolytes. Transitioning from redox to capacitive behavior ultimately leads to a loss in energy density. To maintain redox behavior during Li^+^ intercalation over multiple cycles, one strategy could be to increase ion desolvation during intercalation, for example, using water-in-salt electrolytes (WISEs).^49^ By reducing the number of intercalated water molecules per Li^+^ ion, WISEs might also prevent water-induced oxidation and extend the electrochemical stability window, thereby overcoming the narrow potential window typically imposed by conventional aqueous electrolytes. Furthermore, because WISEs enable complete deintercalation without degrading the MXene structure, they can also enhance overall device performance. In organic electrolytes, Xie *et al.*^48^ reported that Li^+^ intercalation decreases the Ti oxidation state, while deintercalation increases it, as expected for standard battery materials. This opposite behavior compared to aqueous electrolytes arises because organic solvents solvate Li^+^ ions much more weakly than water.^46^ As a result, desolvated Li^+^ ions strongly bind with the electron rich –O terminations, promoting Ti reduction. WISEs enable a mechanism similar to organic electrolytes, but with the added benefit of being water-based and therefore inherently safer.

Notably, while our study focuses on individual MXene flakes, flakes up to ∼35 layers were examined. Across this thickness range (2–35 layers), significant differences in local Ti oxidation state changes were observed, linking nanoscale mechanisms to bulk behavior. In 0.1 M H_2_SO_4_, thin flakes exhibit some irreversible oxidation, while thicker flakes exhibit reduction during spontaneous and electrochemical H^+^ intercalation (∼0.1 e^−^ per atom) with specific capacitance falling in the same range as for high surface area bulk electrodes. In the acidic electrolyte, the accessibility of redox sites is maintained for thick flakes and low water-induced oxidation is observed. High surface area bulk electrodes are therefore beneficial in such electrolyte.^32^

In 0.1 M Li_2_SO_4_, thin flakes exhibit uniform oxidation with localized hotspots, whereas thicker flakes show edge to basal plane gradients, with overall smaller average changes. As per the literature, bulk electrodes show purely capacitive behaviour which could be related to the smaller changes seen for thick flakes. Moreover, independent of the flake thickness, Ti oxidation induced by Li^+^ intercalation occurs mainly during the first 2–3 cycles, after which the oxidation state stabilizes. This reflects a transition from redox to capacitive behavior, as reported for bulk electrodes,^6^ which here is observed for few-layered flakes. The pre-intercalation of the bulk electrode with alkali ion has also been shown as a strategy to increase capacitance in an acidic electrolyte.^50^ Indeed, in this case, the reversible oxidation of Ti atoms may increase the number of electrons exchanged per redox site. Overall, these thickness-dependent experiments provide a bridge from single-flake studies to bulk electrodes and allow connecting nanoscale phenomena to the performance of stacked MXene films.

## Conclusions

In summary, *in situ* nanoscale chemical imaging of individual Ti_3_C_2_T_x_ MXene flakes reveals fundamentally distinct pseudocapacitive electrochemical charge storage mechanisms in acidic or neutral aqueous electrolytes. Proton intercalation induces a reduction in the Ti oxidation state, whereas Li^+^ intercalation leads to an increase in the Ti oxidation state. This divergence stems from their different chemical interaction with the MXene surface: co-intercalated water and partially desolvated Li^+^ ions prompt surface oxidation, whereas protons effectively screen the electron rich oxygen terminations. In the Li^+^ electrolyte, the system evolves from faradaic to capacitive behavior with continued cycling, likely due to the saturation of the active surface sites and the diminished role of redox processes. Furthermore, local variations in the surface chemistry at the nanoscale are evidenced, suggesting that the MXene surface chemistry uniformity, defect density and thickness homogeneity play a role in their pseudocapacitive properties. This work lays the groundwork for understanding charge transfer processes at the nanoscale and provides a basis for future research aimed at optimizing pseudocapacitive energy storage devices.

## Experimental section

### Materials

For the synthesis of MXenes, hydrofluoric acid (HF, 48.5–51%, ACS reagent, Sigma Aldrich), hydrochloric acid (HCl, 36.5–38%, Fisher Chemical), and lithium chloride (LiCl, 99%, ACROS Organics) were used. Sulphuric acid (H_2_SO_4_, ROTH, 95%), lithium sulphate (Li_2_SO_4_, ≥99.9%), and lithium chloride (LiCl, 99%, ACROS Organics) were used for the electrolyte. All electrolytes were prepared with ultrapure water (>18.2 MΩ, Millipore).

### MXene synthesis

Ti_3_C_2_T_x_ MXenes are synthesized by etching the Al layer from the Ti_3_AlC_2_ MAX phase (provided by Jesus Gonzalez-Julian, CNRS) using a mixture of HF, HCl and ultrapure water in the volume ratio of 1 : 6 : 3, following the procedure reported by Mathis *et al.*^24^ 1 g of MAX phase is stirred in 25 mL of acid mixture for 24 hours at 35 °C in an oil bath in a fume hood. The obtained multilayer MXenes are washed multiple times in ultrapure water until the pH reaches between 5 and 7. To delaminate the multilayer MXenes, they are then immersed in 50 mL of aqueous 0.5 M LiCl solution and stirred for 18 hours at room temperature. After that, the intercalated multilayer MXene sheets are washed multiple times with ultrapure water (150 mL each time). The first two washes are done by centrifuging at 3500 rpm for 10 min. The third wash is done for 60 min to remove excess Li^+^. By the fourth wash, the supernatant becomes thick and is collected as delaminated Ti_3_C_2_T_x_ MXenes. To increase the stability and shelf life of the sample, the supernatant is finally concentrated by centrifuging it at 3500 rpm in 60 mL tubes and the concentrated settled MXenes at the bottom are collected. The storage vial is additionally flushed with Ar and stored at 5 °C until the experiment to reduce risks of oxidation.

### Characterization

X-ray diffraction patterns of the MAX phase and synthesized MXenes (presented in Fig. S1) were measured using a Bruker D8 Advance X-ray diffractometer with a Cu Kα source, a step size of 0.03°, and a dwell period of 2 s. The DIFFRAC.EVA software (version 3.1, Bruker) was used to index the diffraction patterns in compliance with the ICDD-PDF-2 database. A free-standing film prepared from 5 mL of 0.3 mg mL^−1^ MXene dispersion was used for this measurement. The MAX phase was measured in powder form.

The morphology of the MXene flakes was examined using scanning electron microscopy (SEM). The SEM micrographs presented in Fig. S2 were recorded on a Zeiss MERLIN microscope, at an acceleration voltage of 3 kV. For these measurements, the samples were prepared by drop casting diluted (4 µL mL^−1^) MXene colloidal suspension onto an ITO substrate.

### Scanning transmission X-ray microscopy

The measurements were performed at the high vacuum scanning transmission X-ray microscopy endstation ‘MYSTIIC’ of the UE-48 undulator beamline at the BESSY II synchrotron operated by HZB. This microscope provides a spectral resolution of <0.1 eV. Ti L-edge spectra over the full energy range (450–475 eV) were acquired, based on which high resolution images with 30 nm steps were captured at 6 energies that include 4 L_2_ and L_3_ peak energies along with pre- and post-edge images at 450 and 475 eV, respectively, allowing us to grasp most of the chemical information with a higher spatial and temporal resolution. For every measurement, bare regions near individual MXene flakes allowed us to measure the incident flux (*I*_0_) essential for optical density calculations.

The *in situ* measurements were performed using a dedicatedly developed transmission cell by NORCADA. The schematic in Fig. S4a shows the path of the X-ray beam, where monochromatic X-rays coming from the synchrotron are directed to the zone plate which focuses the beam onto the sample. The order sorting aperture blocks the higher order light, allowing only 1st order light to reach the sample. The beam is raster scanned across the sample, and the transmitted X-rays are collected in transmission using a photon detector.

For sample preparation, the MXene aqueous solution was diluted to a concentration of 0.1 mg mL^−1^. 1 µL of diluted solution was then immediately drop cast on the 200 × 80 µm^2^ Au/carbon-coated SiN_*x*_ window of an electrochemical chip, as presented in Fig. S4c, with precise and accurate placement of the sample using NORCADA shadow masks and jigs. Allowing the sample to dry at room temperature resulted in the deposition of well-separated monolayer and few-layer MXene flakes suitable for STXM characterization.

To minimize beam effects, which we recently evidenced during point scan measurements on MXenes in an aqueous environment,^20^ we implemented the following strategies:

(1) Reduced the dwell time to 1 ms per pixel. (*vs*. *ca.* 1000 ms for point scans).

(2) Energy-selective imaging instead of acquiring full spectra, focusing on pre-edge, L_3_ and L_2_ t_2g_/e_g_ peaks, and the post-edge (no point scans were performed)

(3) Kept the beam shutters off when not acquiring data to avoid unnecessary exposure.

(4) Use of a flow cell, which continuously removes radiolytic species generated during X-ray exposure, preserving the intrinsic chemistry of the flakes.

These measures ensure that the chemical information obtained reflects the intrinsic state of the flakes rather than beam-induced artifacts.

## Author contributions

Funding acquisition for the project was carried out by T. P. Conceptualization of the idea was done by T. P. and N. S. Methodology, including the design and commissioning of the *in situ* cell, was developed by T. P., N. S., L. G., P. B., and M. W. Beamtime planning for the synchrotron-based experiments was handled by T. P., N. S., and L. G. Experiments were performed by N. S., L. G., P. B., F. A., A. W., Z. D., and M. L., under the supervision of T. P. and M. W. Data analysis was conducted by N. S. and L. G. and supervised by T. P. Manuscript writing was done by N. S., under the supervision of L. G. and T. P.

## Conflicts of interest

There are no conflicts of interest.

## Supplementary Material

EE-019-D5EE05809K-s001

## Data Availability

The data that support the findings of this study are openly available in Zenodo at https://doi.org/10.5281/zenodo.17670135, reference number 17670135. The data supporting this article have been included as part of the supplementary information (SI). Supplementary information is available. See DOI: https://doi.org/10.1039/d5ee05809k.
