# Supplementary material for: Nanoscale chemical imaging of pseudocapacitive charge storage in MXenes
Source: Energy Environ Sci. 2025 Dec 16;19(2):680–90. doi: 10.1039/d5ee05809k (PMC12767768; doi:10.1039/d5ee05809k)
Supplement: EE-019-D5EE05809K-s001 [file EE-019-D5EE05809K-s001.pdf]

Supplementary Information (SI) for Energy & Environmental Science.
This journal is © The Royal Society of Chemistry 2025

Nanoscale chemical imaging of pseudocapacitive charge storage in MXene

Authors: Namrata Sharma[1,2], Louis Godeffroy[1], Peer Bärmann[1], Faidra Amargianou[1,2], Andreas Weisser[1,2], Zoé Dessoliers[1,3], Mailis Lounasvuori[1], Markus Weigand[1], Tristan Petit[1*]

Affiliation:

[1]Helmholtz-Zentrum Berlin für Materialien und Energie GmbH, Albert-Einstein-Straße 15, 12489 Berlin, Germany

[2]Faculty of Mathematics and Natural Sciences, TU-Berlin, Hardenbergstraße 36, 10623 Berlin, Germany

[3]Faculty III - Process Sciences, TU-Berlin, Straße des 17. Juni 135, 10623 Berlin, Germany

*Corresponding author. Email: Tristan.Petit@helmholtz-belin.de

Contents

1. Material characterization ...3

Fig. S1 | XRD patterns ...3

Fig. S2 | SEM images ...3

Fig. S3 | XPS survey of delaminated Ti_3C_2Tx MXenes in (a) ...4

2. In situ scanning transmission X-ray microscopy experiments ...5

Fig. S4 | Schematic of ...5

1. Supplementary Discussion 1: spatial and chemical resolution estimation ...6

Fig. S5 | Spatial resolution estimation. ...7

2. Supplementary Discussion 2: Estimation of the Ti oxidation state from the Ti L_2 e_g/t_{2g} peak intensity ratio ...8

Fig. S6 | Ti oxidation state estimation ...9

Supplementary Table 1 ...9

3. Supplementary data and image analysis: spontaneous H^+ intercalation ...10

Fig. S8 | L_2 e_g/t_{2g} peak intensity ratio heatmaps ...11

Fig. S9| L_2 e_g (H2SO4) / e_g (H2O) peak intensity ratio heatmaps. ...11

4. Supplementary data and image analysis: electrochemical H^+ intercalation ...12

Fig. S10 |Flake thickness estimation and electrochemical data in 0.1 M H_2SO_4 ...12

Fig. S11 | Thickness dependent Ti oxidation state comparison ...13

Fig. S12 | L_2 e_g/t_{2g} peak intensity ratio heat maps ...14

Fig. S13| L_2 e_g (-0.76 V) / e_g(-0.13 V) peak intensity ratio heatmaps ...14

5. Supplementary data and image analysis: spontaneous Li^+ intercalation ...15

Fig. S14 | Flake thickness estimation ...15

Fig. S15 | L_2 e_g/t_{2g} peak intensity ratio heat maps ...15

Fig. S16 | $Ti_3C_2T_X$ MXenes in LiCl ...16

Fig. S17 | Thickness dependent XAS in 0.1 M Li_2SO_4 ...16

Fig. S18 | L_2 e_g (Li2SO4) / e_g(H2O) peak intensity ratio heatmaps ...16

6. Supplementary data and image analysis: electrochemical Li^+ intercalation ...16

Fig. S19 | Electrochemical Li+ intercalation and deintercalation. ...17

Fig. S20 | Electrochemical data in 0.1 M Li_2SO_4. ...18

Fig. S21| L_2 eg peak intensity ratio for Li intercalation and deintercalation. ...18

Fig. S22 | Electrochemical oxidation of ...19

Fig. S23 | L_2 e_g/t_{2g} peak intensity ratio heat maps ...19

7. Supplementary Discussion 3: active pixel estimation for all data sets ...19

Supplementary Table 2 ...20

8. Supplementary Discussion 4: Specific capacitance estimation ...21

1. Material characterization

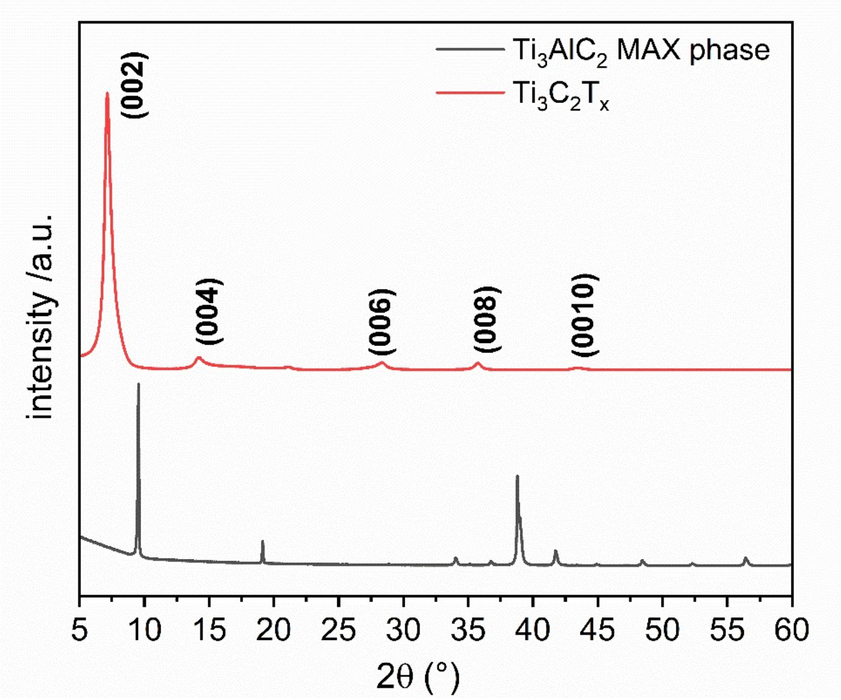

Fig. S1 | XRD patterns of MAX phase and synthesized $Ti_3C_2T_x$ MXenes.

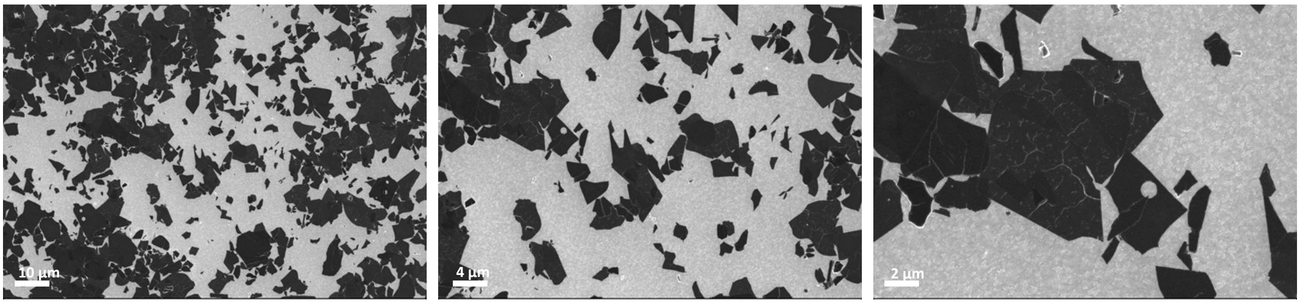

Fig. S2 | SEM images of synthesized $Ti_3C_2T_x$ MXenes on ITO substrate**.**

a)

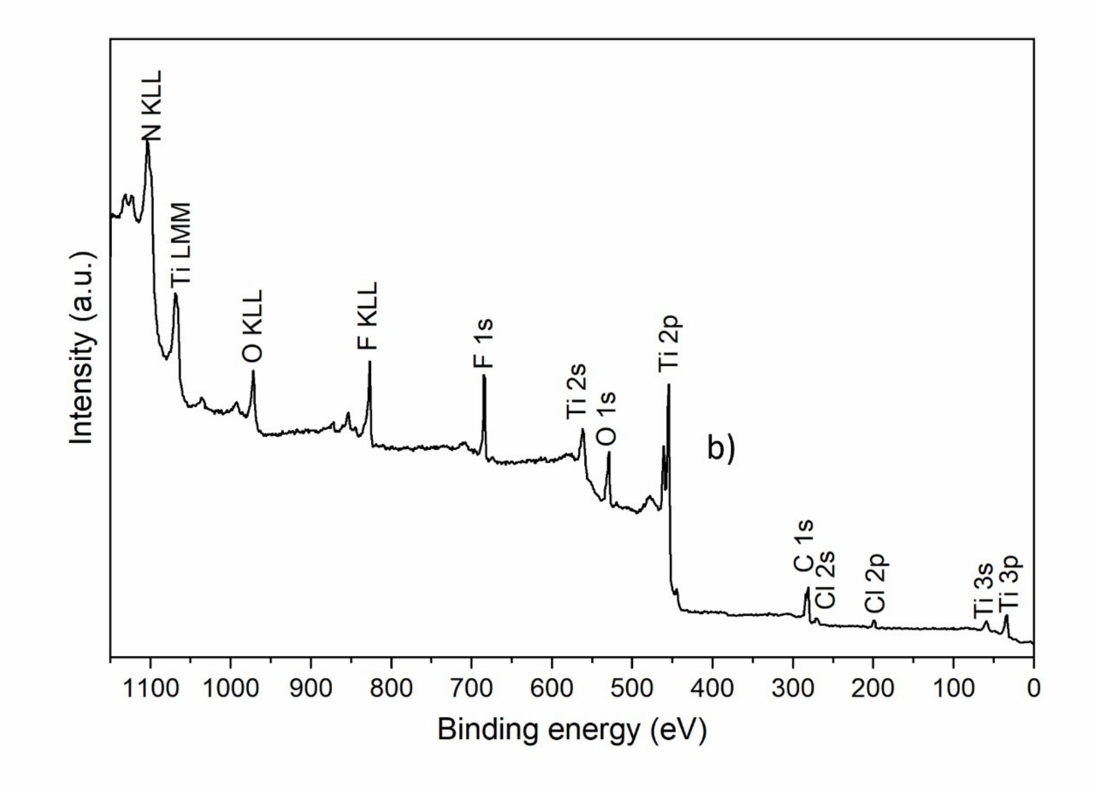

XPS quantification

b)

	Al Kalpha XPS						
Ti3C2Tx	Name	Position	RSF	IMFP	Raw Area	Area/(RSF*T*MFP)	%At Conc (Full region)
	F-Ti	688,52	1	1,524	10125	6644	12,3
	Ti-O	529,55	0,733	1,670	4295,33	3509	6,5
	Ti-O'	531,05	0,733	1,670	5057,48	4132	7,6
	#NAME?	532,9	0,733	1,670	151,01	123	0,2
	H2O_OR	534	0,733	1,670	269,02	220	0,4
	Ti1	455	2,077	1,730	18833,14	5242	9,7
	Ti2	456,29	2,077	1,730	7211,52	2007	3,7
	Ti3	457,3	2,077	1,730	2010,1	559	1,0
	Ti4	459	2,077	1,730	2333,4	649	1,2
	Ti1'	460,92	2,077	1,730	9416,57	2621	4,9
	Ti2'	462,21	2,077	1,730	3605,76	1004	1,9
	Ti3'	463,22	2,077	1,730	1005,05	280	0,5
	Ti4'	464,92	2,077	1,730	2204,83	614	1,1
	C-C/H	284,87	0,314	1,868	9110,58	15536	28,8
	C-O	286,44	0,314	1,868	5468,92	9326	17,3
	COO/C=O	288,5	0,314	1,868	547,09	933	1,7
	Cl 3/2	198,89	0,954	1,937	771,29	417	0,8
	Cl 1/2	200,49	0,954	1,937	385,64	209	0,4

Element	At%
F	12,3
O	14,8
Ti	24,0
C	47,7
Cl	1,2
total	100,0

Fig. S3 | XPS survey of delaminated Ti_3C_2Tx MXenes in (a) with quantification in (b)

2. In situ scanning transmission X-ray microscopy experiments

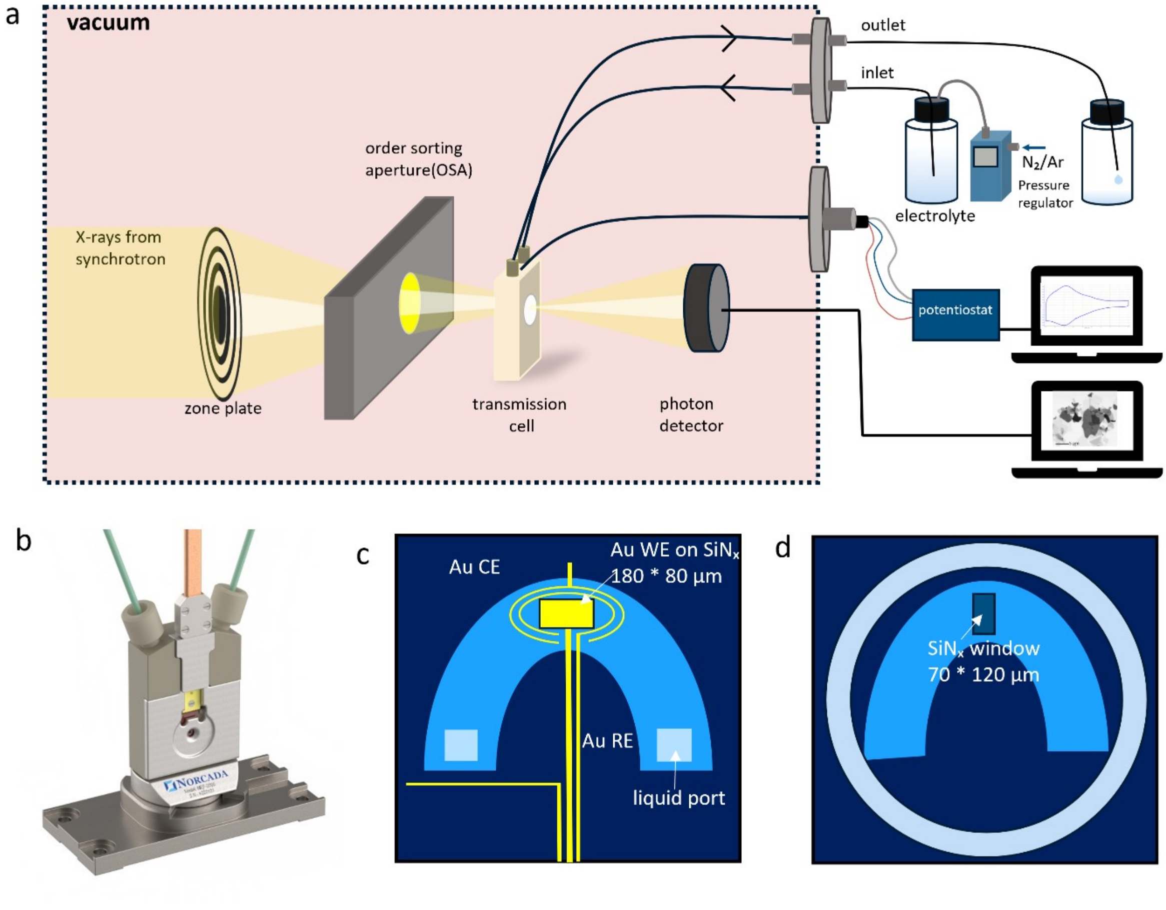

Fig. S4 | Schematic of (a) the in-situ STXM experimental station, (b) the transmission cell, (c) the electrochemical bottom chip, and (d) the top chip.

The corrosion resistant transmission cell (Fig. S4b) has a fully removable and replaceable PEEK (polyetheretherketone) body and fluidic tubing which allows measurement in sulfuric acid at low pH of 1. The MXenes are drop casted on an electrochemical chip presented in Fig. S4c. The MXenes and the liquid layer are sandwiched between two silicon nitride (SiN_x)

windows (Supplementary Fig 3c, d). The bottom chip embeds 3 electrodes: the working electrode (Au/C) is on the 200 x 80 μm² SiN_x window where the MXene flakes are drop casted, encircled by the reference (Au/Pt) and counter electrodes (Au/Pt). Two liquid ports serving as inlet and outlet for continuous flow of electrolyte are included in the bottom chip. The top chip (Fig. S4d) has an optimized 70 x 120 μm² SiN_x window to allow transmission of the X-rays across the entire cell, and a proprietary gasket to make the cell vacuum tight. The SiN_x window on the top chip is designed with a larger vertical and a smaller horizontal dimension relative to the bottom chip to allow for the best alignment and control the liquid layer thickness. While bringing the two chips together, the distance between the two SiN_x windows is set to lower than 10 μm to minimise attenuation of the soft X-rays. The chips are placed in a dedicated recessed space in the peak body of the cell. This spacer internally connects the liquid ports on the bottom chip to the inlet and outlet of the cell body.

The liquid flow in the electrochemical cell is controlled by a pressure-driven pump (Fluigent microfluidic flow controller). For all measurements, the electrolyte was degassed with Ar. N_2 was used to control the pump and a flow rate of 80-100 μL/min was maintained. Constant flow of electrolyte throughout measurement minimises interaction between radiolytic species and the sample. It also removes any gas released during the electrochemical reactions, preventing the formation of gas bubbles which would disrupt the measurement. The SiN_x windows are found to withstand a high flow rate of up to 800 μL/min. The electrodes of the transmission electrochemical cell are connected to a Biologic SP-300 potentiostat with the ultralow current option that allows measurements down to pA range.

1. Supplementary Discussion 1: spatial and chemical resolution estimation

The spatial resolution of STXM is defined as the minimum distance between two distinguishable structures, which can be estimated by plotting OD absorption profile.[12] To estimate the spatial resolution, we use a high resolution OD image acquired at the L_2 e_g peak energy (463 eV) with a pixel size of 30 nm (Fig. S5a). To quantitatively assess the spatial resolution, the absorption profile across the edge of a representative bilayer flake and a 5-layer flake was analyzed (Fig. S5). The measured OD contrast between the flake and the surrounding background is approximately 0.12, and 0.25, consistent with a 2-3 and a 5 layers flake, respectively.[7]

The OD profiles are fitted using a sigmoid step function, which captures the gradual transition from the substrate to the flake region. The width of this transition (10-90% rise distance) represents the convolution of the true structural interface (which for thin flakes like these ones we can safely assume is a perfect rectangular step) with the microscope's point-spread function. For the bilayer flake, the fitted step width corresponds to 2.48 pixels, indicating that approximately 2-3 pixels are required to distinguish the flake from the background for a thin flake (~75 nm) For the 5-layer flake, the step width corresponds to 1.66 pixels or ~49 nm (Fig. S5 c, d). Hence, based on these results, the resolution is necessarily <49 nm. The higher step width for the bilayer flake could be due to a lower signal-to-noise ratio but could also indicate a more defective edge.

It is worth noting that the image was acquired in situ in the presence of water. In this configuration, the effective resolution may be slightly reduced compared to vacuum

measurements due to X-ray scattering in the liquid layer. Consequently, the measured step width (~75 nm) represents an upper limit for a bilayer flake, combining both instrumental and environmental broadening. While the nominal STXM resolution may be below 50 nm under ideal vacuum conditions, in-situ measurements of thin flakes yield an effective resolution no better than ~75 nm for bilayers layer, however around 50 nm for 5 layer and above.

During image analysis, the STXM data acquired at 30 nm pixel size were averaged 2×2 to reduce noise (Fig. 4, main text). Subsequently, heat maps representing chemical changes at the nanoscale were generated. This filtering reduces the effective chemical resolution from ~75 nm to approximately 150 nm, as the averaging combines local variations over total 4 pixels. For transparency, raw ratio heat maps without filtering are provided in the Supporting Information (Fig. S18), allowing readers to evaluate unprocessed nanoscale features.

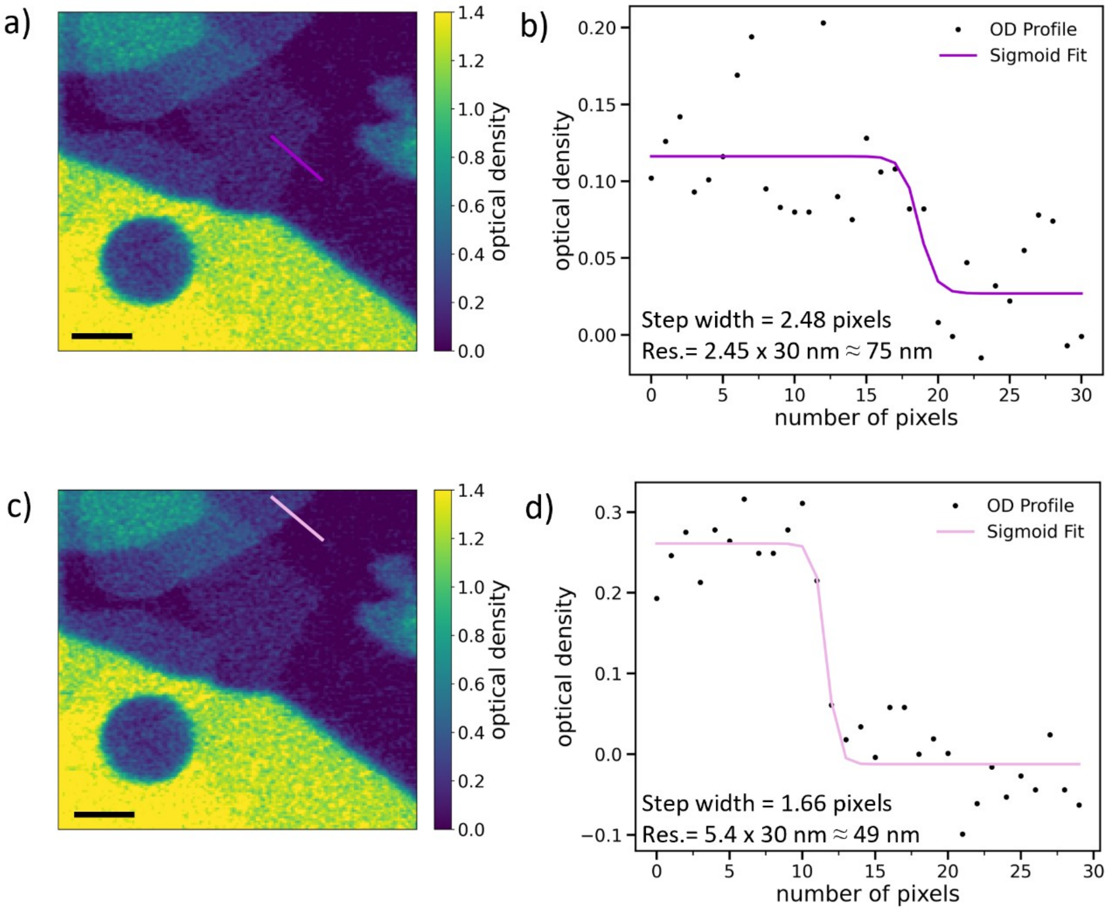

Fig. S5 | Spatial resolution estimation. a) High-resolution (30 nm pixel size) STXM image at the Ti L_2 e_g peak energy (463 eV) in water, (b) corresponding absorption profile across purple line (2-layer flake) in (a), (d) absorption profile across pink line (5-layer flake) in (c). Scale bar: 1 µm.

2. Supplementary Discussion 2: Estimation of the Ti oxidation state from the Ti L_2 e_g/t_{2g} peak intensity ratio

XAS at the metal L-edge is a powerful technique to estimate the oxidation state of transition metals. Similar approach as the one described below has also been implemented to quantify the Fe and Cu oxidation state in $CuFeS_2$ by Yao *et al.*[3] Freitas *et al.*[4] published detailed guidelines for Mo oxidation state extrapolation from Mo L-edge XAS measurements using commercial Mo compounds as reference.[4] The population of the e_g unoccupied electronic state is a good marker of the electronic structure and has been found to vary with the Ti oxidation state.[5] Although the Ti oxidation state in $Ti_3C_2T_x$ MXenes was already estimated from Ti K-edge XAS using titanium oxides as references,[6] we are not aware of a previous report estimating the Ti oxidation state from Ti L-edge XAS. Indeed, the quantitative estimation of the oxidation requires transmission XAS measurements, because fluorescence and electron-yield XAS measurements are subject to artefacts that may change the relative intensity of the L_2 and L_3 peak components.[7]

Here, to quantify the Ti oxidation state changes, the mean Ti L_2 e_g/t_{2g} peak intensity ratio is compared. Since no significant shifts in energy positions are observed for L-edge spectra, the change in oxidation state is usually evaluated based on L_2/L_3 peak intensity. This has been implemented before for estimating the oxidation state of other transition metals like Ni, Mn, V etc.[3,4] At the same time, higher e_g compared to t_{2g} was also reported with increasing Ni oxidation state. We have adopted a similar concept for estimating Ti oxidation state. A direct correlation between Ti L_2 e_g/t_{2g} peak intensity ratio with Ti oxidation state was reported before by our group.[2,8] An increase in the ratio implies a greater number of unoccupied e_g orbitals and thus an increase in oxidation state. Assuming a linear dependence between the peak ratio and Ti oxidation state, the oxidation state variations can be roughly estimated using anatase TiO_2 and pristine MXene as references.

Due to an uneven distribution of surface functional groups (-O, -OH, -F, -Cl) depending on the synthesis route, it is complicated to calculate or estimate the exact oxidation state of Ti in pristine $Ti_3C_2T_x$ MXenes. However, based on a previous estimation done by Lukatskaya *et al.* of Ti oxidation state through K-edge XAS measurements, 2.4 is used as reference average oxidation state of Ti for pristine $Ti_3C_2T_x$ MXenes.[6]

The authors used wet chemical etching for synthesis, similar to our work. Although they employed in situ HF generation from LiF + HCl as opposed to direct HF: HCl: H_2O mixture in our work (see Methods), both methods produce MXenes with mixed -O, -OH, and -F surface terminations. Despite differences in safety, reaction rate, or etching control of the two etching methods, the underlying chemistry and resulting MXene structure remain fundamentally comparable.[9]

For the extrapolation, Anatase TiO_2 nanoribbons previously measured by STXM are used as a reference Ti(+IV) oxidation state for the extrapolation of the Ti-L_2 e_g/t_{2g} ratio.[10,11] Other titanium oxides (e.g., TiO, Ti_2O_3) were not considered because they are difficult to measure in transmission mode due to the thickness of commercially available powders. Note that previous reports of XAS recorded in total electron yield mode cannot be used for quantitative analysis because the signal is not directly proportional to the pure absorption.[2]

Ti L_2 e_g/t_{2g} peak intensity ratio is calculated for pristine MXene, the spectrum of which is presented in Fig. S6a. The histograms generated from the ratio images are used for estimating the mean ratio presented throughout the article. The mean ratio is obtained by fitting the histogram using a Voigt function. Fig. S6b, c and Supplementary Table 1 show the mean ratio values for $Ti_3C_2T_x$ MXene under different measurement conditions (pristine, in water, in 0.1 M H_2SO_4). These ratio values are used for Ti oxidation state estimation in Fig. S6c with error bars calculated using equations (S1), (S2) and (S3) below:

$$\sigma_{mean} = \frac{FWHM_{Voigt}}{2.355\sqrt{N}} \quad \text{(S1)}$$

$$FWHM_{Voigt} \approx 0.5346 \times (2\gamma) + \sqrt{0.2166 \times (2\gamma)^2 + (2.3548 \times \sigma)^2} \quad \text{(S2)}$$

$$\text{error} = 3 * \sigma_{mean} \quad \text{(S3)}$$

where σ_{mean} is the error on the mean, $FWHM_{Voigt}$ the full width at half maximum of the Voigt distribution, N the number of pixels considered for the histogram, γ the gamma factor of the Lorentzian part, and σ the standard deviation of the Gaussian part.

Fig. S6c shows the extrapolation curve. The sources, ratio and extrapolated oxidation states with errors are presented in Supplementary Table 1. This approach has been used throughout this work for Ti oxidation state estimation.

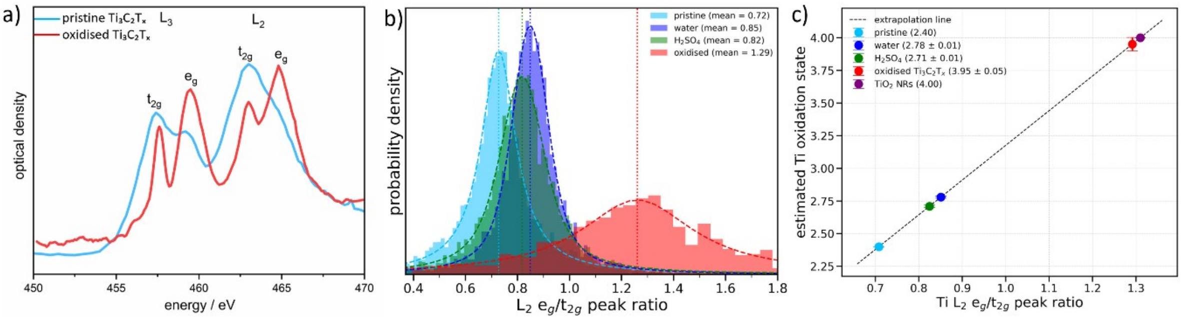

Fig. S6 | Ti oxidation state estimation. (a) XAS spectrum of pristine (blue) and oxidized (red) $Ti_3C_2T_x$ MXenes. The spectrum of pristine MXenes was used as reference for oxidation state estimation, whereas that of oxidized MXene is shown for comparison. (b) Pixel intensity histograms of the Ti L_2 e_g/t_{2g} peak ratio heat maps measured in different conditions. (c) Corresponding mean peak ratio values and extrapolated oxidation states.

Supplementary Table 1 | Ti oxidation state estimation using Ti L_2 e_g/t_{2g} peak ratios.

Sample condition	Ti L_2 e_g/t_{2g} peak ratio	Known Ti oxidation state	Estimated Ti oxidation state
Pristine $Ti_3C_2T_x$	0.72 (Fig. 5a, b)	2.4 [6]	
Ti NRs	1.31 [11]	4.0	
$Ti_3C_2T_x$ in water	0.85		2.78 ± 0.01
$Ti_3C_2T_x$ in H_2SO_4	0.82		2.71 ± 0.01
$Ti_3C_2T_x$ oxidised	1.29		3.95 ± 0.05

To further validate the extrapolation approach, we measured the Ti-L edge spectra of chemically oxidized $Ti_3C_2T_x$ MXenes (Fig. S6 - red color). The Ti-L_2 e_g/t_{2g} ratio for these oxidized MXenes is close to that of TiO_2 and falls well on the linear extrapolation line, supporting the validity of this method. Further, acknowledging the inherent uncertainty of our measurements and conversion method, we explicitly note that the Ti oxidation states reported are *estimated* values.

3. Supplementary data and image analysis: spontaneous H^+ intercalation

The exact thickness of a flake can be estimated from the optical density of the XAS spectrum.[2] The thickness of the clusters in Fig. S7a is estimated through the optical density spectra presented in Fig. S7b. Fig. S7c presents the decrease in Ti L_2 e_g/t_{2g} intensity ratio with increasing flake thickness in 0.1 M H_2SO_4. We observed that it remained largely constant across the entire region of interest in both environments, suggesting rapid proton diffusion throughout the flakes in H_2SO_4 irrespective of thickness and overlapping. Only the thinnest flakes (estimated at 2-3 layers) have a slightly higher peak ratio than rest, but it still decreases in the same way in H_2SO_4.

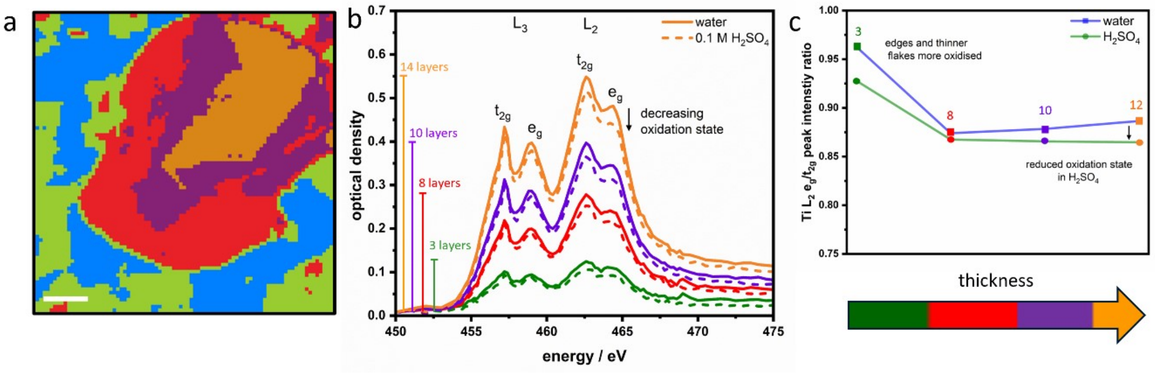

Fig. S7 | Flake thickness estimation. (a) Thickness-based clusters calculated from the data presented in Fig. 1. (b) Corresponding optical density spectra at the Ti L-edge. (c) Comparative average Ti L_2 e_g/t_{2g} peak intensity ratios in water and 0.1 M H_2SO_4 for the different clusters in (a). Scale bar: 1 μm.

The XAS spectra presented in Fig. 1d are averaged over the flakes shown in Fig. S8. Introducing water into the cell caused the SiN_x window to shift, thereby altering the transmission area and restricting a direct comparison between spectra measured in air and in water. Nonetheless, since the flowing liquid uniformly wets the window, the shift does not influence the spectral features themselves. The pixel intensity histograms presented in Fig. 1e are generated from L_2 e_g/t_{2g} peak ratio heat maps presented in Fig. S8.

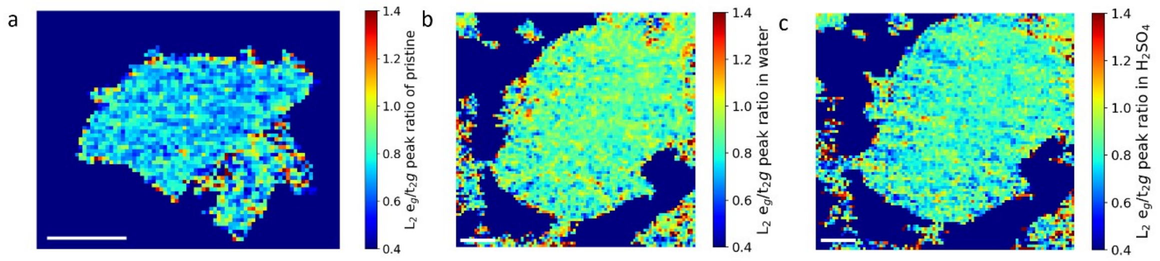

Fig. S8 | L_2 e_g/t_{2g} peak intensity ratio heatmaps normalized with pre-edge image (a) in their pristine state (in air), (b) in water, and (c) in 0.1 M H_2SO_4. Scale bars: 1 μm

For better visual, uniform filters are used before calculating the ratio image to reduce noise (Fig. S9). The uniform filter is an image processing tool used for smoothing or denoising data by applying a local averaging operation. It works by replacing each pixel value with the average of its surrounding pixels within a defined neighborhood, specified by the size parameter (here 4). This averaging window, often square (e.g., 4×4 or 5×5), moves across the image, producing a new image where local intensity variations are reduced. As a result, the uniform filter helps suppress high-frequency noise and minor fluctuations, making it particularly useful for visual clarity in scientific imaging, such as optical density maps. Same approach has been used for ratio images in each data set.

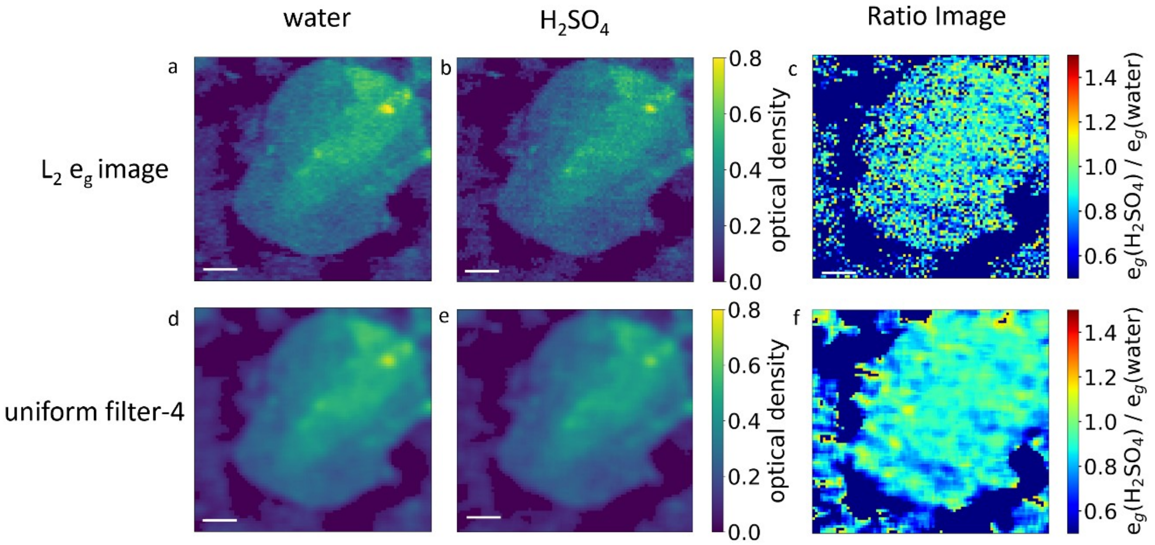

Fig. S9| L_2 e_g (H2SO4) / e_g (H2O) peak intensity ratio heatmaps. STXM images at the Ti L_2 e_g peak energy (463 eV) (a, b) before and (d, e) after applied uniform filter, in water and in 0.1 M H_2SO_4, respectively. Ti L_2 e_g ratio heat maps between 0.1 M H_2SO_4 and water using (c) unfiltered and (f) filtered images. Scale bars: 1 μm.

4. Supplementary data and image analysis: electrochemical H^+ intercalation

The thickness of the clusters in Fig. S10b is estimated through the optical density spectra presented in Fig. S10e. Fig. S10f presents the Ti L_2 e_g/t_{2g} intensity ratio with flake thickness in 0.1 M H_2SO_4 at ocv and applied -0.76 V. We observed that it remained largely constant across the entire region of interest at both potentials, suggesting rapid proton diffusion throughout the flakes in H_2SO_4 irrespective of thickness and overlapping(therefore refereed as thick overlapping flake in main). Only the thinnest flakes (estimated at 2-3 layers, called bilayer in main) doesn't show any change H_2SO_4. This is likely because they exist in an oxidized state and cannot be reduced under the applied potential.

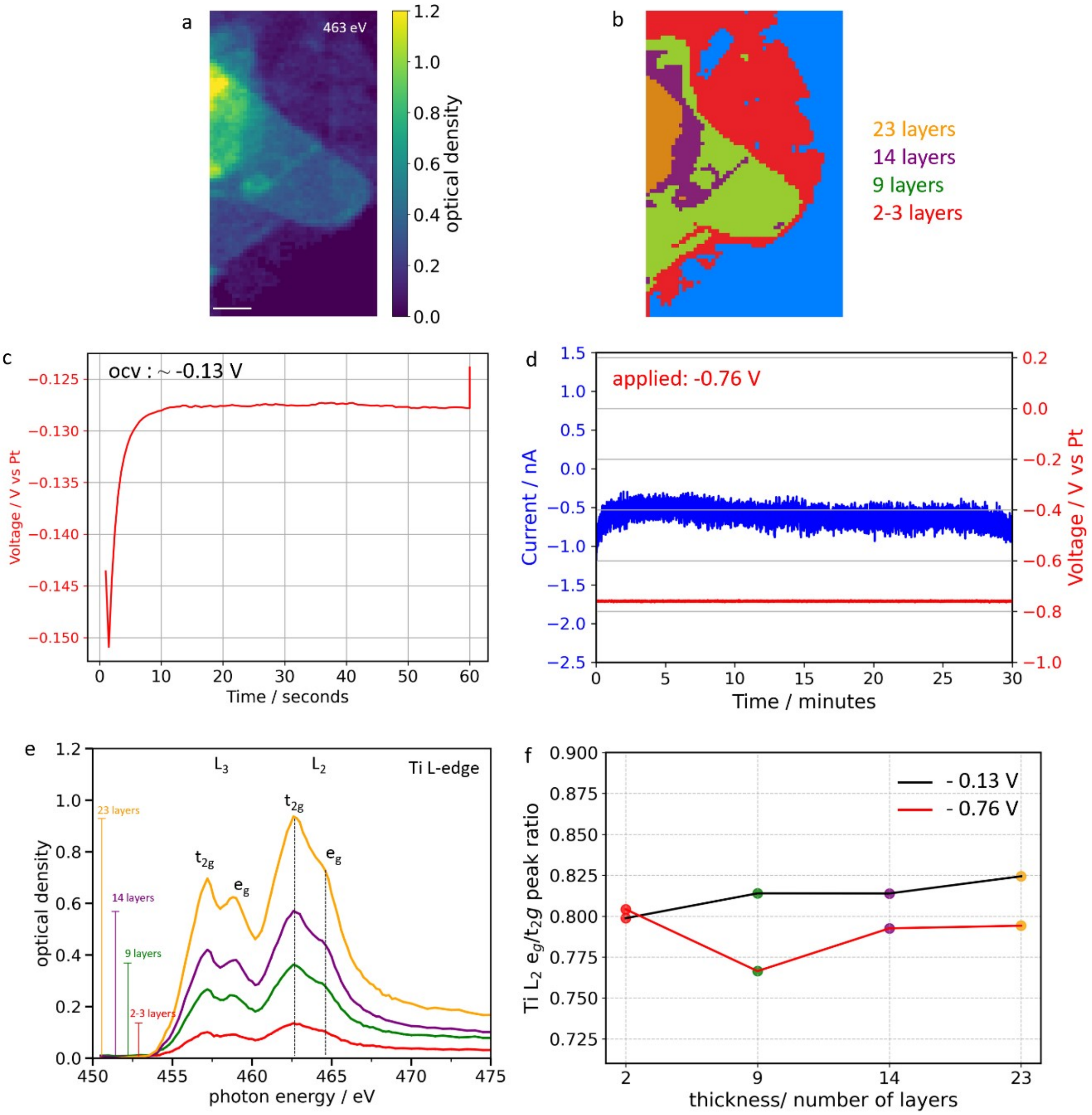

Fig. S10 |Flake thickness estimation and electrochemical data in 0.1 M H_2SO_4. (a) STXM of $Ti_3C_2T_x$ MXenes at 463 eV, (b) Thickness-based clusters calculated from the data presented on (a). (b) OCV measurement performed in 0.1 M H_2SO_4 on the C chip discussed in Fig. 2 and flakes in (a). (d) Chronoamperometry at -0.76 V vs Pt in 0.1 M H_2SO_4 measured on the same

chip.(e) corresponding optical density spectra at the Ti L-edge to clusters in (b). (f) comparative average Ti L_2 e_g/t_{2g} peak intensity ratios at ocv: -0.13 V and applied -0.76 V for the different clusters in (b). Scale bar: 1 µm.

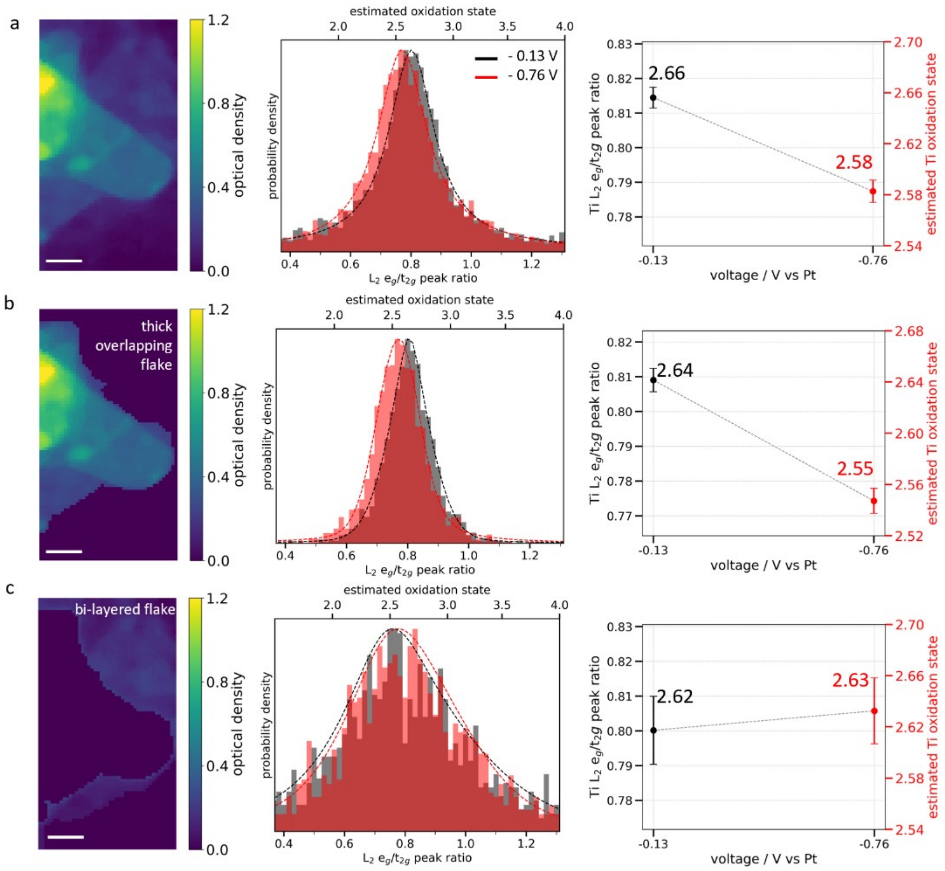

Fig. S11 | Thickness dependent Ti oxidation state comparison. STXM image of $Ti_3C_2T_x$ MXenes at 463 eV, distribution of Ti L_2 e_g/t_{2g} peak ratio for the different potentials with estimated average Ti oxidation state for (a) the full area, (b) only the thick overlapping flake, and (c) only the bi-layered flakes. Scale bars: 1 µm.

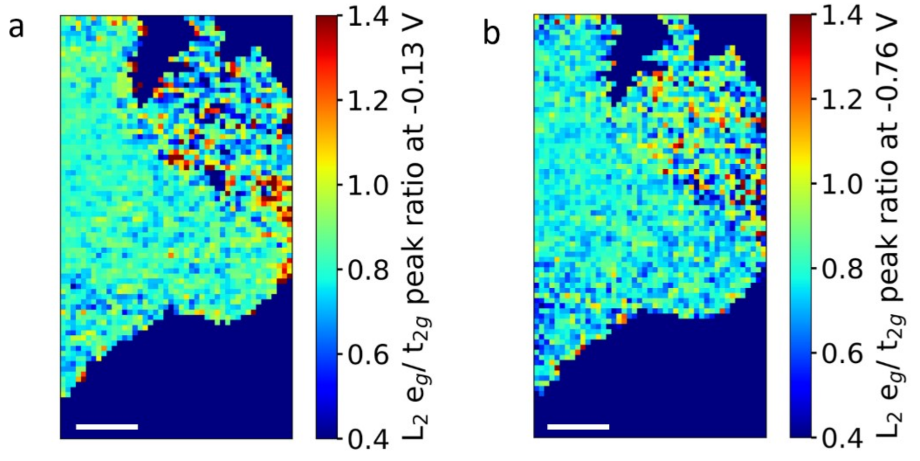

Fig. S12 | L_2 e_g/t_{2g} peak intensity ratio heat maps normalized with pre-edge image at (a) ocv (~-0.13 V) and (b) applied -0.76 V vs Pt. Scale bars: 1 μm.

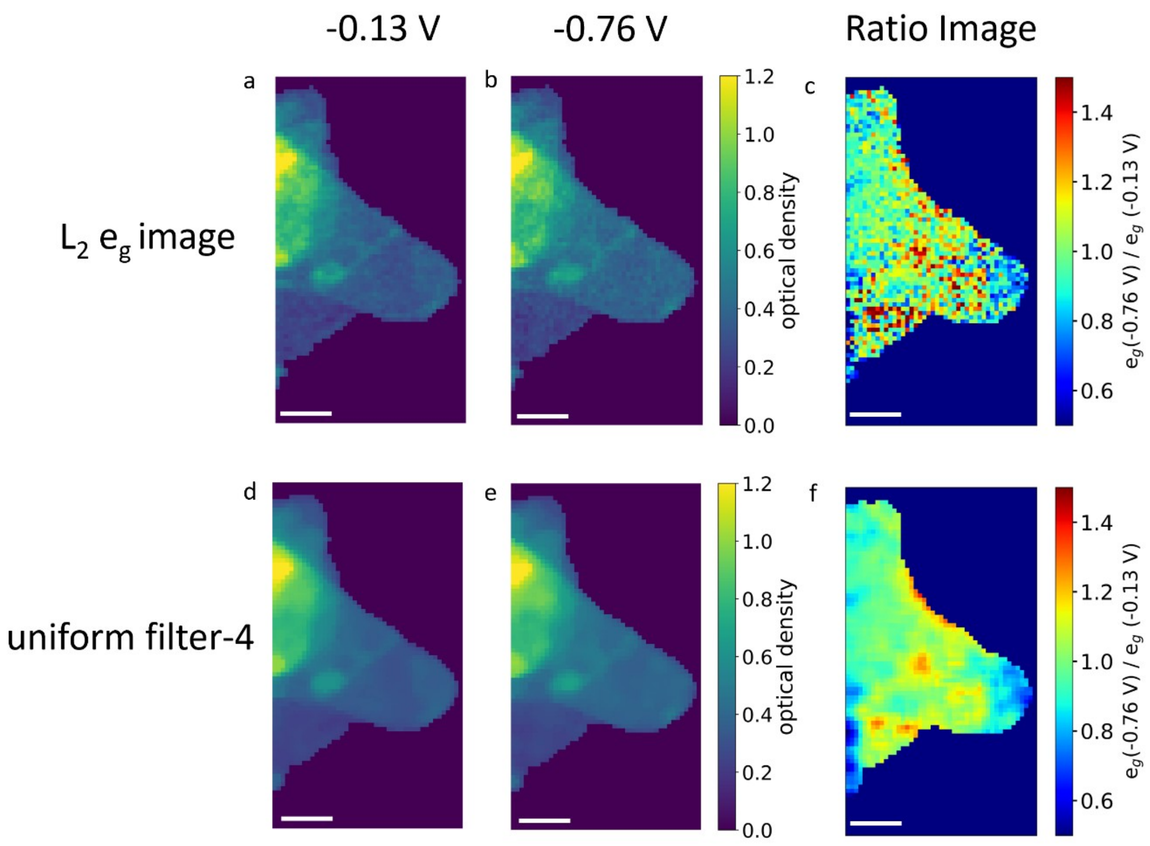

Fig. S13| L_2 e_g (-0.76 V) / e_g(-0.13 V) peak intensity ratio heatmaps. STXM images at the Ti L_2 e_g peak energy (463 eV) in 0.1 M H_2SO_4 (a, b) before and (d, e) after applied uniform filter, at -0.13 V and -0.76 V vs Pt, respectively. Ti L_2 e_g ratio heat maps between -0.76 V and -0.13 V using (c) unfiltered and (f) filtered images. Scale bars: 1 μm.

5. Supplementary data and image analysis: spontaneous Li^+ intercalation

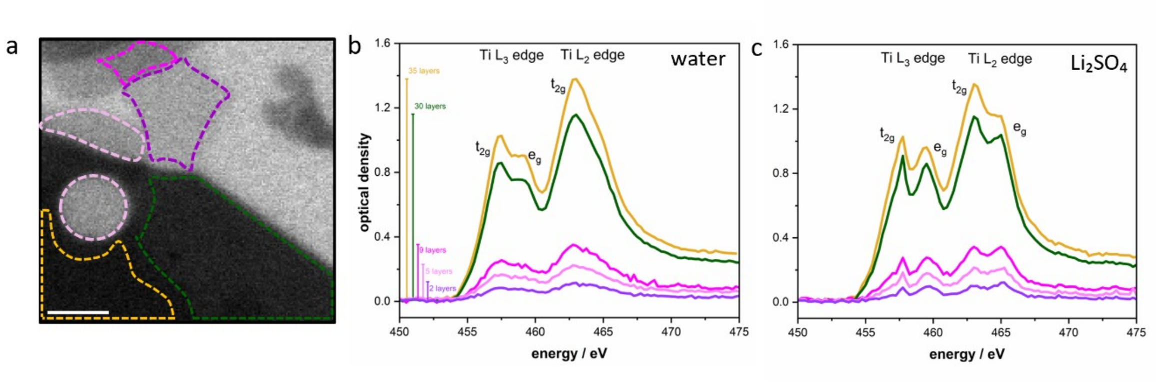

Fig. S14 | Flake thickness estimation. (a) STXM image of $Ti_3C_2T_X$ MXenes at the Ti L-edge (463 eV) in water. Highlighted regions correspond to 2-layer (purple dotted), 5-layer (pink dotted), 8-layer (magenta dotted), 30-layer (green dotted) and 35-layer (mustard dotted) flakes. Corresponding optical density spectra (b) in water and (c) in 0.1 M Li_2SO_4 electrolyte along with the thickness estimation of the flakes. Scale bar: 1 μm.

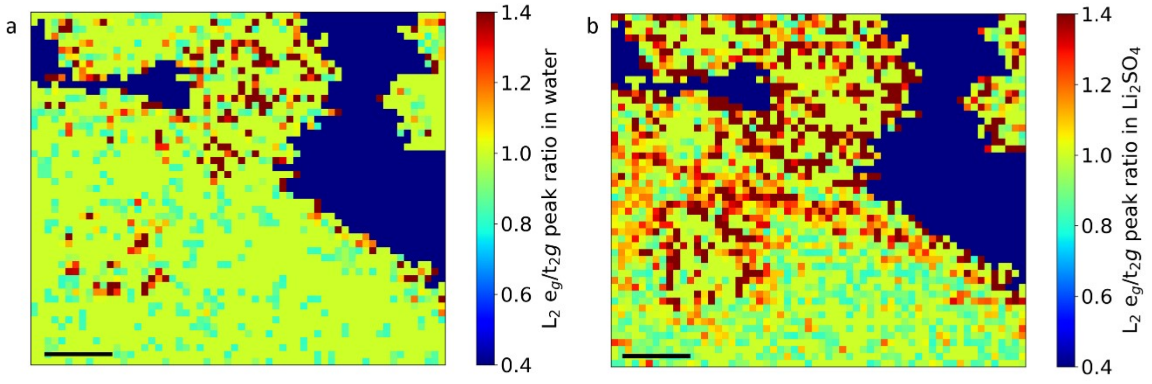

Fig. S15 | L_2 e_g/t_{2g} peak intensity ratio heat maps normalized with pre-edge image obtained (a) in water and (b) in 0.1 M Li_2SO_4. Scale bars: 1 μm.

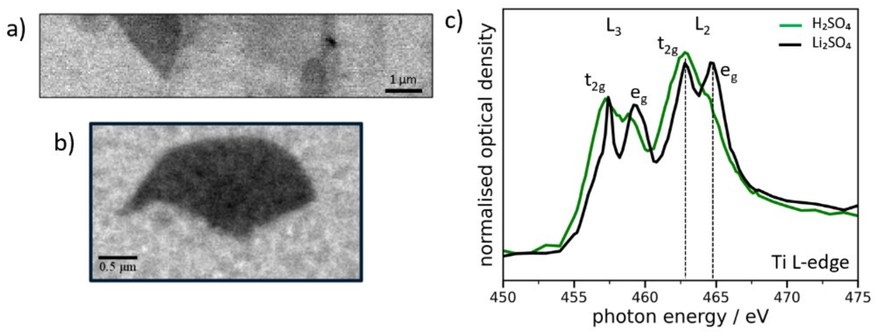

Fig. S16 | $Ti_3C_2T_X$ MXenes in LiCl. STXM image at the Ti L-edge (463 eV) in (a) 0.1 M H_2SO_4, (b) 0.1 M LiCl. Comparative average Ti L-edge XAS spectra of the flake in 0.1 M H_2SO_4 (green) and 0.1 M LiCl (black).

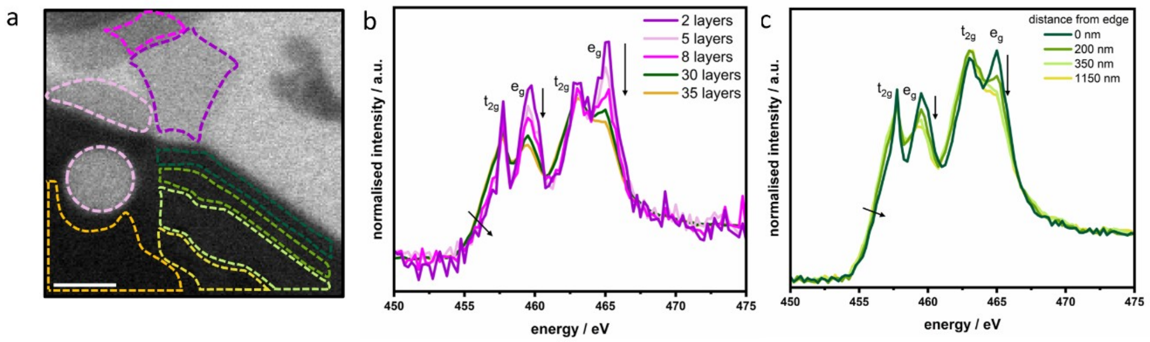

Fig. S17 | Thickness dependent XAS in 0.1 M Li_2SO_4. (a) STXM image at the Ti L-edge (463 eV) in water, along with highlighted areas of interest (dotted) for tracking ion intercalation based on thickness as well as from edge to basal plane. Corresponding XAS spectra are presented in (b) and (c), respectively. Scale bar: 1 μm.

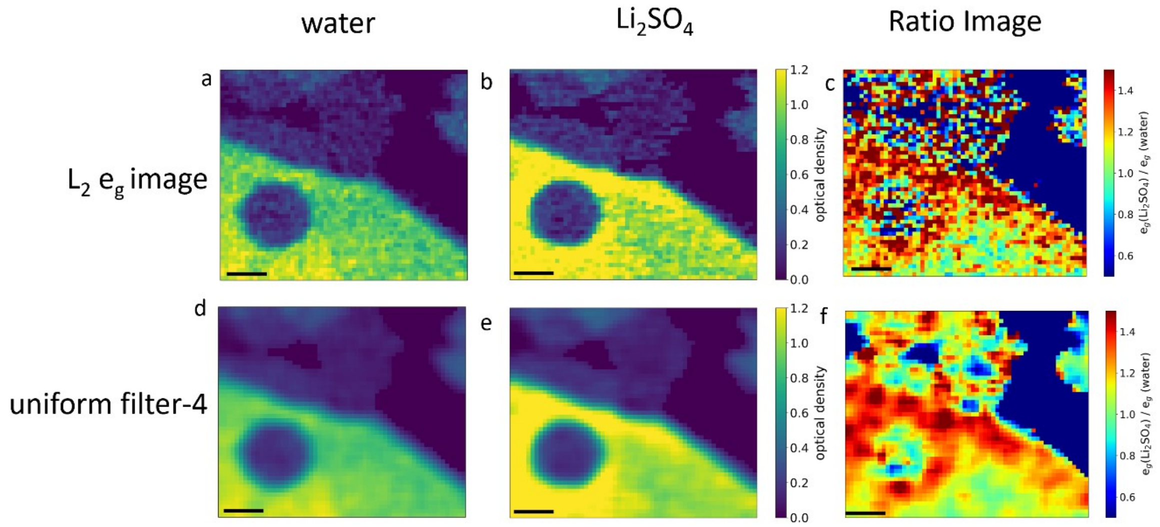

Fig. S18 | L_2 e_g (Li2SO4) / e_g(H2O) peak intensity ratio heatmaps. STXM images at the Ti L_2 e_g peak energy (463 eV) in water and 0.1 M Li_2SO_4 (a, b) before and (d, e) after applied uniform filter. Ti L_2 e_g ratio heat maps between 0.1 M Li_2SO_4 and water using (c) unfiltered and (f) filtered images. Scale bars: 1 μm.

6. Supplementary data and image analysis: electrochemical Li^+ intercalation

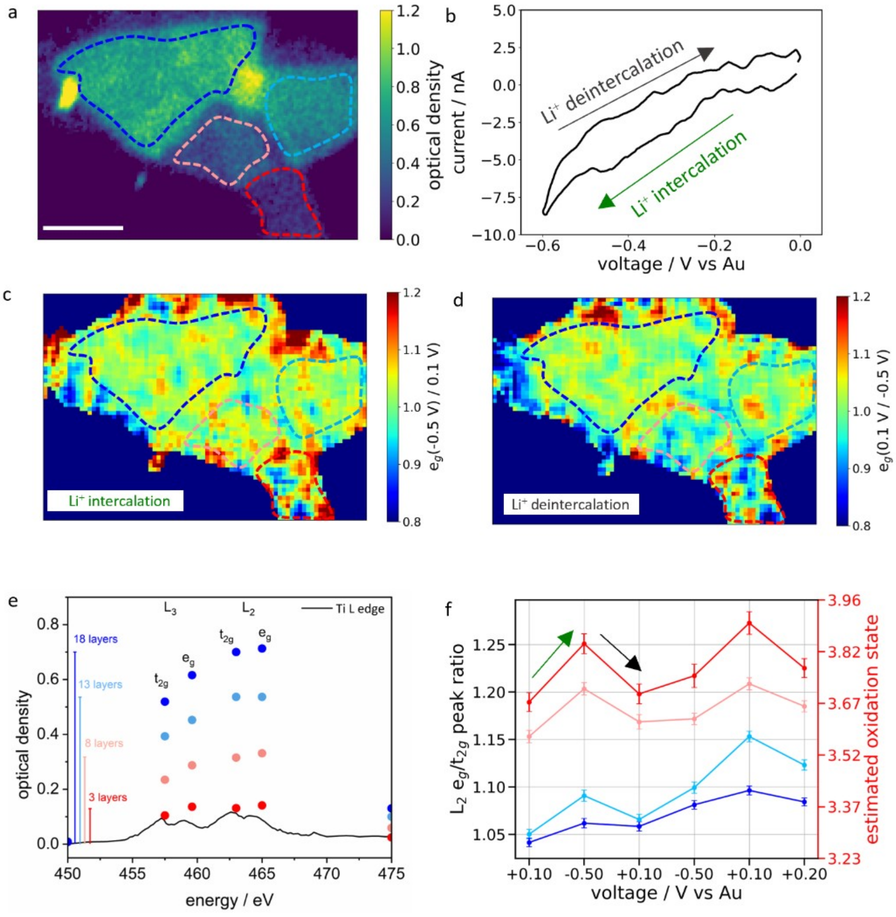

Fig. S19 | Electrochemical Li+ intercalation and deintercalation. (a) STXM image of $Ti_3C_2T_x$ MXene flakes at the Ti L-edge (463 eV) in 0.1 M Li_2SO_4 electrolyte. Highlighted regions correspond to 3-layer (red dotted), 8-layer (pink dotted), 13-layer (cyan dotted), and 18-layer (blue dotted) flakes. (b) Cyclic voltammogram measured in 0.1 M Li_2SO_4 on the same Au chip. (c, d) L_2 e_g peak ratio heat maps representing Li^+ ion intercalation and deintercalation, respectively. (e) XAS spectra of different flakes in (a), along with thickness estimation. (f) Comparative Ti L_2 e_g/t_{2g} peak intensity ratios in the charged and discharged states over two consecutive cycles. Scale bars: 1 μm.

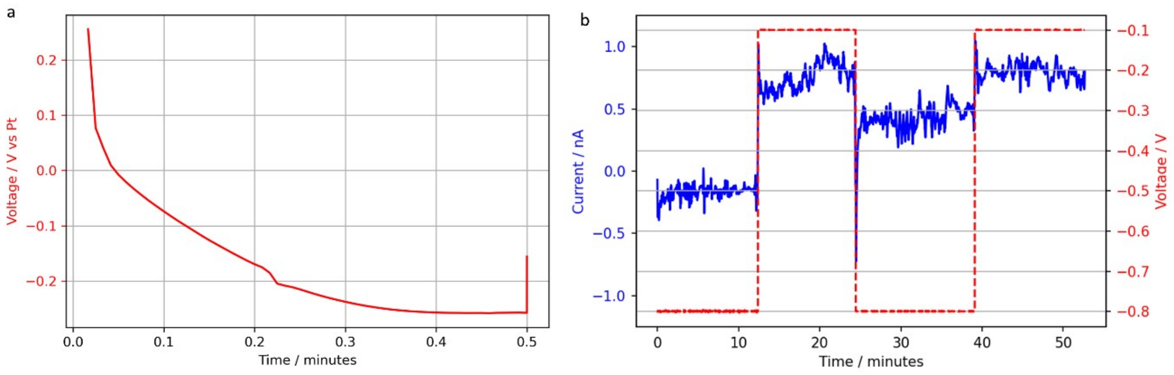

Fig. S20 | Electrochemical data in 0.1 M Li_2SO_4. (a) OCV measurement performed in 0.1 M Li_2SO_4 on the Au chip discussed in Fig. 4. (b) Multi-step chronoamperometry measured between -0.8 V and -0.1 V vs Pt in 0.1 M Li_2SO_4 electrolyte on the same chip.

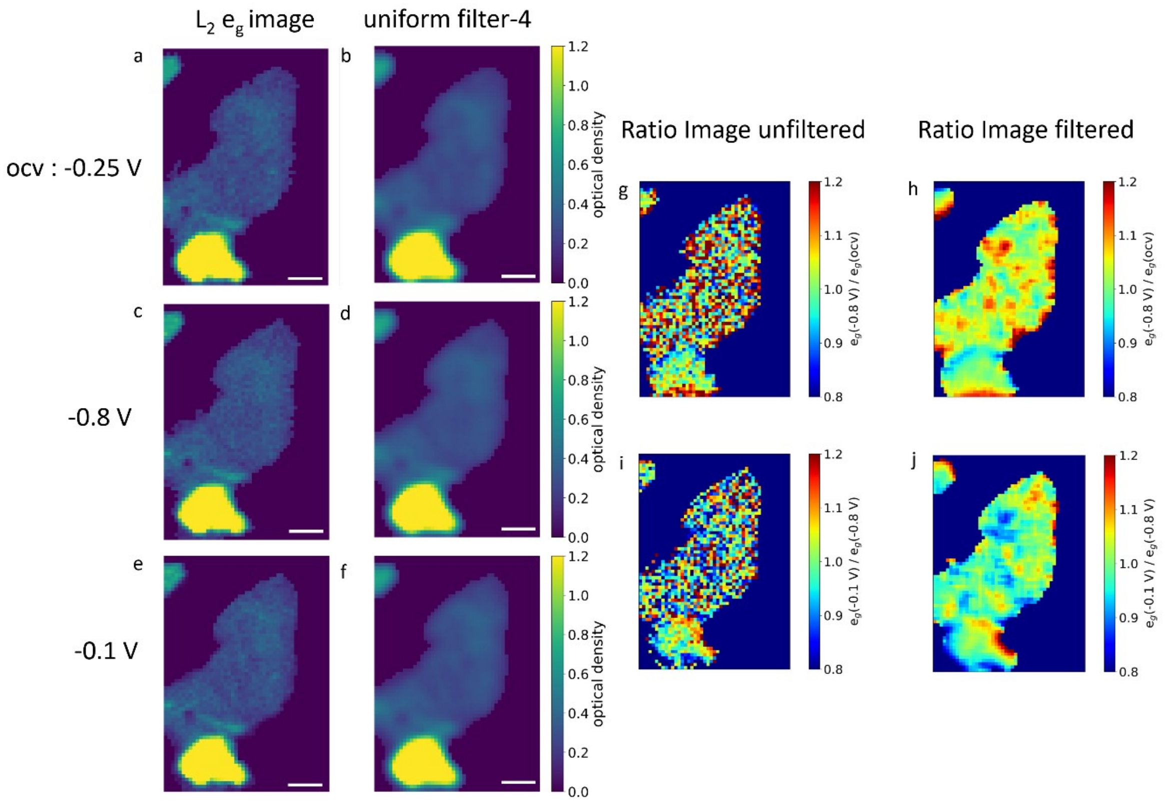

Fig. S21| L_2 eg peak intensity ratio for Li intercalation and deintercalation. STXM images at the Ti L_2 e_g peak energy without and with applied uniform filter (a, b) at ocv (-0.25 V), (c, d) at applied -0.8 V vs Pt, and (e, f) at applied -0.1 V, all in 0.1 M Li_2SO_4. L_2 e_g peak ratio heat maps calculated using the raw and filtered images, (g, h) between -0.8 V and the ocv and (i, j) between -0.1 V and -0.8 V. Scale bars: 1 µm.

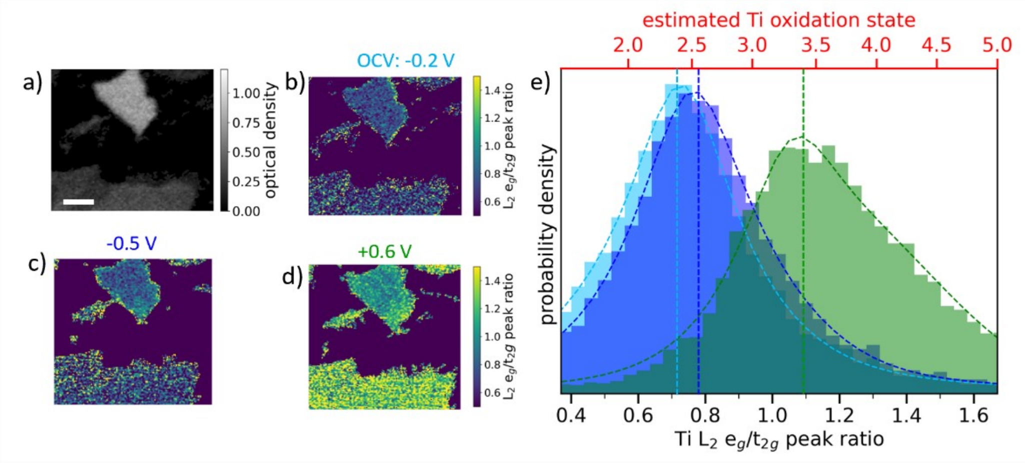

Fig. S22 | Electrochemical oxidation of $Ti_3C_2T_x$ in Li_2SO_4. (a) STXM image of $Ti_3C_2T_x$ MXene flakes at the Ti L-edge (463 eV) in 0.1 M Li_2SO_4 electrolyte. Ti L_2 e_g/t_{2g} peak ratio heat map at (b) OCV (0.04 V vs Au), (c) -0.5 V, and (d) +0.6 V. (e) Corresponding pixel intensity histograms along with estimated average Ti oxidation state. Scale bar: 1 µm.

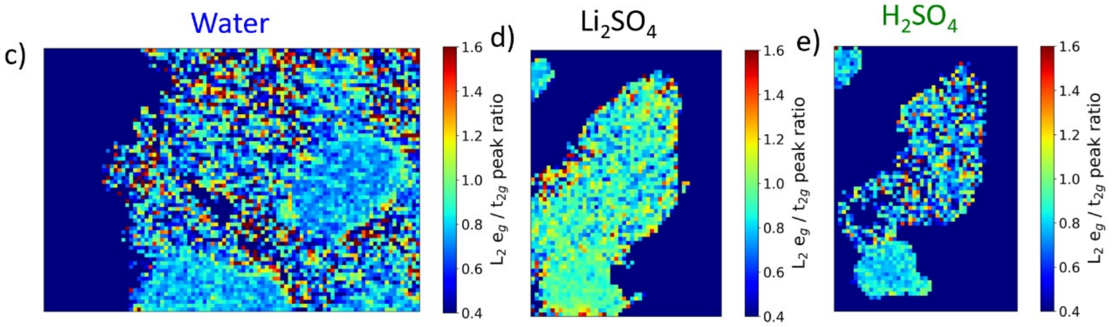

Fig. S23 | L_2 e_g/t_{2g} peak intensity ratio heat maps normalized with pre-edge image obtained (a) in water, (b) in 0.1 M Li_2SO_4, and (c) in 0.1 M H_2SO_4. Scale bars: 1 µm.

7. Supplementary Discussion 3: active pixel estimation for all data sets

The non-uniform Ti oxidation state distribution is highlighted in the e_g/e_g ratio heatmaps and thickness-based comparisons across all data sets (Fig. 1-4, S9, S13, S18, S19 & S23). For quantitative analysis, we have estimated the percentage of active pixels (pixels showing change) for both thin (OD < 0.35, ~9 layers and below) and thick flake (OD> 0.35, above ~9 layers) in two electrolytes (0.1 M H_2SO_4 and Li_2SO_4) for both spontaneous and electrochemical (de)intercalation conditions. Pixels showing an e_g/e_g ratio ≥ 1 are considered active sites for oxidation, while pixels with a ratio< 1 are considered active sites for reduction. A summary of the results is presented in the table below.

For spontaneous intercalation, where the MXene flakes were first exposed to water and then to the electrolyte, the number of active sites is higher in thick flakes compared to thin flakes. Overall, the number of active sites is high approximately around 70-90% in both electrolytes.

After spontaneous intercalation, a negative potential was applied that promoted further ion intercalation. On average, the number of active sites decreased for both thin and thick flakes, but the trend remained the same in H_2SO_4, with thick flakes having more active sites than thin ones. In Li_2SO_4, the number of active pixels remained roughly the same for both thin and thick flakes. Additionally, when a positive potential was applied to de-intercalate Li ions, the number of active pixels further decreased by about 10-15%, likely due to irreversible oxidation caused by a combination of surface lithiation and water-induced oxidation.

Overall, the average number of active sites during spontaneous intercalation is 70-90%, which can be explained by the surface termination distribution. The MXenes used have -F terminations of around 30-40%, as quantified by XPS (Fig.S2b), which act as inactive sites. Therefore, the fraction of inactive sites can be attributed to fluorine terminations.

After spontaneous intercalation, applying a negative potential in both electrolytes further drives ion intercalation. Since some sites are already protonated or lithiated due to spontaneous intercalation, the average number of active sites decreases by roughly 10%.

We also observe a clear relationship between flake thickness and active sites: thick flakes have more active sites than thin flakes. This is because thin flakes are more vulnerable and prone to oxidation in water already, which reduces the number of active sites compared to thick flakes.

Supplementary Table 2 | Active pixel estimation from Ti L_2 e_g/e_g ratios.

Spontaneous/electrochemical intercalation	Flake thickness	Percentage of active areas $\frac{number\ of\ pixels\ in\ active\ areas}{total\ pixels} *$
**Spontaneous intercalation**		
0.1 M H_2SO_4	Thin (9 layers)	~78%
	Thick (9+ layers)	~91%
0.1 M Li_2SO_4	Thin	~73%
	thick	~93%
**Electrochemical intercalation**		
0.1 M H_2SO_4 (H^+ intercalation)	Thin	~64%
	Thick	~88%
0.1 M Li_2SO_4 (Li^+ intercalation)	Thin	~77%
	Thick	~74%
0.1 M Li_2SO_4 (Li^+ de-intercalation)	Thin	~60%
	Thick	~61%

8. Supplementary Discussion 4: Specific capacitance estimation

"The specific capacitance (C_s) can be estimated from the charge stored by a single $Ti_3C_2T_x$ MXene flake (Q_{flake}) and mass of that flake (m_{flake})[12], which can then be compared to bulk electrodes, using formula

$$C_s = \frac{Q_{\text{flake}}}{m_{\text{flake}}\,\Delta V} \tag{S4}$$

ΔV=voltage window

Q_{flake} is estimated based on the number of titanium atoms in the flake (N_{Ti}) and the change in oxidation state (per Ti atom, ΔOS) presented in the manuscript/determined from the STXM experiments.

Step 1: Unit cell area

The lateral/projected area of the flake A_{flake} is estimated by counting the number of pixels on the masked flake and multiplying it with area of pixel, and unit cell area ($A_{\text{unit cell}}$) is calculated considering a lattice parameter of a = 3.03 Å for the hexagonal cell of $Ti_3C_2T_x$.[13]

$$A_{\text{unit cell}} = \frac{\sqrt{3}}{2}a^2 = 7.95 \times 10^{-20}\ \text{m}^2 \tag{S5}$$

Step 2: Number of Ti atoms in a monolayer

Each unit cell contains 6 Ti atoms ($N_{\text{Ti/unit cell}} = 6$)

The total number of Ti atoms in a monolayer flake is therefore

$$N_{\text{Ti}} = \frac{A_{\text{flake}}}{A_{\text{unit cell}}} \cdot N_{\text{Ti/unit cell}} \tag{S6}$$

Step 3: Total electrons transferred

The total number of electrons transferred is

$$N_e = N_{\text{Ti}} \cdot \Delta_{\text{OS}}. \tag{S7}$$

Δ_{OS}. = *Ti electron transfer /atom (from e_g/t_{2g} ratio)*

Step 4: Charge per monolayer flake

The charge stored by a monolayer flake is then calculated as

$$Q_{\text{monolayer}} = N_e \cdot e \tag{S8}$$

e = elementary charge (1.602 x 10^{-19} C)

Step 5: Total charge for few layered flake

$$Q_{\text{flake}} = Q_{\text{monolayer}} \cdot N_{\text{layers}} \qquad \text{(S9)}$$

N_{layers}= *Number of layers in the flake (estimated from optical density)*

Step 6: Mass of the flake

The mass of the few-layered flake (m_{flake}) is estimated using the specific surface area ($SSA_{1\ layer}$) of a $Ti_3C_2T_x$ MXene monolayer, calculated based on the formula proposed by Cabré *et al.* The exact nature of the surface terminations $T_x = O_{1.9}F_{1.5}Cl_{0.2}$ was determined by XPS (Fig. S2b) :

$$m_{\text{flake}} = \frac{A_{\text{flake}}}{SSA_{1\ \text{layer}}} \cdot N_{\text{layers}} \qquad \text{(S10)}$$

$$SSA_{1\ layer} = \frac{area\ of\ monolayer}{mass\ of\ Ti_3C_2O_{1.9}F_{1.5}Cl_{0.2}} \qquad \text{(S11)}$$

$$= \frac{\frac{nm^2}{12\ unit\ cells}}{\frac{3.Ti_{MW} + 2..C_{MW} + 1.9.O_{MW} + 1.5F_{MW} + 0.2.Cl_{MW}}{1\ unit\ cell\ \cdot\ N_A}} = 214\ m^2 g^{-1}$$

All parameters can then be extracted from the experimental data and the specific capacitance directly calculated from equation (S4).

Calculations for H_2SO_4

Parameters:

- $A_{\text{flake}} = 8.91 \times 10^{-12}$ m² (Fig. 2b)
- $\Delta_{\text{Ox}} = 0.1$ (section in main: Electrochemical H+ intercalation, page 5)
- $N_{\text{layers}} = 15$ (average flake thickness estimated from Fig. S8)
- ΔV = 0.7V (Fig. 2a)

Specific capacitance in 0.1 M H_2SO_4 ≈ 370 F/g

Calculations for 0.1 M Li_2SO_4

Parameters:

- $A_{\text{flake}} = 1.43 \times 10^{-11}$ m² (Fig. 4a)
- $\Delta_{\text{Ox}} = 0.04$ (section in main: Electrochemical Li+ intercalation, page 9)
- $N_{\text{layers}} = 14$
- ΔV = 0.7V (Fig. 4b)

Specific capacitance in 0.1 M $Li_2SO_4 \approx 147$ F/g

Supplementary References

1 D. A. Shapiro, S. Babin, R. S. Celestre, W. Chao, R. P. Conley, P. Denes, B. Enders, P. Enfedaque, S. James, J. M. Joseph, H. Krishnan, S. Marchesini, K. Muriki, K. Nowrouzi, S. R. Oh, H. Padmore, T. Warwick, L. Yang, V. V Yashchuk, Y.-S. Yu and J. Zhao, *Sci. Adv.*, 2025, **6**, eabc4904.

2 F. Amargianou, P. Bärmann, H. Shao, P. L. Taberna, P. Simon, J. Gonzalez-Julian, M. Weigand and T. Petit, *Small Methods*, 2024, **2400190**, 1–9.

3 Y. Yao, A. Bhargava and R. D. Robinson, *Chem. Mater.*, 2021, **33**, 608–615.

4 A. P. Freitas, R. F. André, C. Poucin, T. K. C. Le, J. Imbao, B. Lassalle-Kaiser and S. Carenco, *J. Phys. Chem. C*, 2021, **125**, 17761–17773.

5 C. X. Kronawitter, J. R. Bakke, D. A. Wheeler, W. C. Wang, C. Chang, B. R. Antoun, J. Z. Zhang, J. Guo, S. F. Bent, S. S. Mao and L. Vayssieres, *Nano Lett.*, 2011, **11**, 3855–3861.

6 M. R. Lukatskaya, S. M. Bak, X. Yu, X. Q. Yang, M. W. Barsoum and Y. Gogotsi, *Adv. Energy Mater.*, 2015, **5**, 2–5.

7 R. Kurian, K. Kunnus, P. Wernet, S. M. Butorin, P. Glatzel and F. M. F. de Groot, *J. Phys. Condens. Matter*, 2012, **24**, 452201.

8 A. Al-Temimy, F. Kronast, M. A. Mawass, K. A. Mazzio, K. Prenger, M. Naguib, T. Petit and S. Raoux, *Appl. Surf. Sci.*, 2020, **530**, 147157.

9 M. Downes, C. E. Shuck, B. McBride, J. Busa and Y. Gogotsi, *Nat. Protoc.*, 2024, **19**, 1807–1834.

10 X. Zhu, A. P. Hitchcock, C. Bittencourt, P. Umek and P. Krüger, *J. Phys. Chem. C*, 2015, **119**, 24192–24200.

11 P. Guttmann, C. Bittencourt, S. Rehbein, P. Umek, X. Ke, G. Van Tendeloo, C. P. Ewels and G. Schneider, *Nat. Photonics*, 2012, **6**, 25–29.

12 M. Brunet Cabré, D. Spurling, P. Martinuz, M. Longhi, C. Schröder, H. Nolan, V. Nicolosi, P. E. Colavita and K. McKelvey, *Nat. Commun.*, 2023, **14**, 1–7.

13 C. B. Cockreham, V. G. Goncharov, E. Hammond-Pereira, M. E. Reece, A. C. Strzelecki, W. Xu, S. R. Saunders, H. Xu, X. Guo and D. Wu, *ACS Appl. Mater. Interfaces*, 2022, **14**, 41542–41554.
